# Diastereoselective Ugi reaction of chiral 1,3-aminoalcohols derived from an organocatalytic Mannich reaction

**DOI:** 10.3762/bjoc.12.15

**Published:** 2016-01-26

**Authors:** Samantha Caputo, Andrea Basso, Lisa Moni, Renata Riva, Valeria Rocca, Luca Banfi

**Affiliations:** 1 Department of Chemistry and Industrial Chemistry, University of Genova, I-16146 Genova, Italy

**Keywords:** aminoalcohols, isocyanides, multicomponent reactions, peptidomimetics, Ugi reaction

## Abstract

Enantiomerically pure β-aminoalcohols, produced through an organocatalytic Mannich reaction, were subjected to an Ugi multicomponent reaction under classical or Lewis acid-promoted conditions with diastereoselectivities ranging from moderate to good. This approach represents a step-economical path to enantiomerically pure, polyfunctionalized peptidomimetics endowed with three stereogenic centers, allowing the introduction of five diversity inputs.

## Findings

Isocyanide-based multicomponent reactions [1–3], such as the Ugi reaction, were demonstrated to be very useful in the rapid assembly of complex drug candidates [4], introducing three to four diversity inputs. Furthermore, a nearly limitless variety of heterocycles can be accessed through post-condensation transformations [5–7], adding only one to two steps to the synthetic sequence. However, the main drawback of the Ugi reaction is the poor stereochemical control that is typically achieved [8–9], which hampers its utilization in the diversity-oriented or target-oriented synthesis of complex chiral peptidomimetics. No efficient asymmetric catalytic classic Ugi reaction has been reported to date (whereas some success was obtained on simpler variants) [10–12]. On the other hand, diastereoselective reactions using at least one chiral component are troublesome. Chiral isocyanides and chiral carboxylic acids invariably afford nearly 1:1 mixtures. α-Chiral aldehydes have a high tendency to racemize/epimerize [13–14] and additionally, no report of valuable diastereocontrol by them has appeared so far. Successful examples of diastereoselective Ugi reactions have been reported only with chiral amines [15–19] or with chiral cyclic imines (Ugi–Joullié reaction) [20–23], although in the latter case, racemization/epimerization can again be an issue in special cases [24]. However, the use of amines as chiral auxiliaries has been seldom exploited in peptidomimetic synthesis [16,25–26] because the need to remove the auxiliary reduces the number of diversity inputs and increases the number of synthetic steps.

From the point of view of atom- and step-economy, the use of chiral amines that are retained in the final products will be more valuable [27]. In this case they are not "chiral auxiliaries" and are not removed after the multicomponent reaction, and they contribute to the diversity of the final products. However, the usefulness of this approach relies on an efficient and diversity-oriented preparation of the required amines in high enantiomeric excess.

Chiral aminoalcohols can be ideal substrates for diastereoselective Ugi reactions: the additional hydroxy group can both help in modulating diastereoselectivity and be employed for post-condensation transformations in order to add further fragments or to form heterocyclic structures. We have previously developed some syntheses of heterocycles through Ugi reactions with 1,2-aminoalcohols followed by nucleophilic substitutions [28], whereas chiral 1,2-aminoalcohols have been proved by Nenajdenko and co-workers to be able to induce good levels of diastereoselectivity in the Ugi reaction [17].

**Scheme 1:**
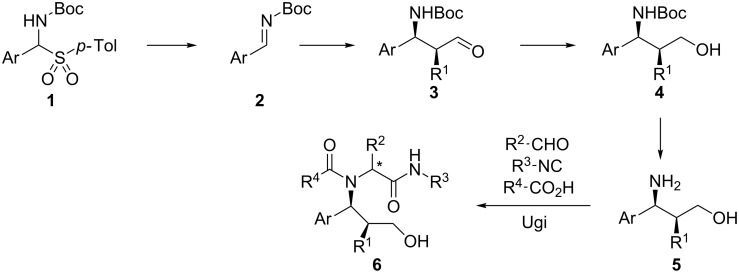
Overall strategy.

Our attention was drawn by 1,3-aminoalcohols of general formula **5** (Scheme 1), which can be obtained by List's organocatalytic Mannich-type reaction of aldehydes with *N*-Boc imines **2** and catalytic L-proline [29–30], followed by reduction of **3** and cleavage of the Boc group.

This short and straightforward synthesis allows the introduction of 2 diversity inputs (R^1^ and Ar), whereas stereochemical diversity can also be explored using D-proline or different, *anti*-selective organocatalysts.

**Scheme 2:**
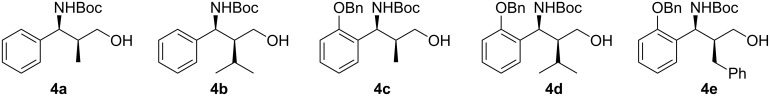
Boc-protected aminoalcohols used as inputs in a diastereoselective Ugi reaction.

We prepared two known carbamoyl sulfones **1** [30–31] and transformed them without isolation of intermediates into a series of five Boc-protected β-aminoalcohols **4a**–**e** (Scheme 2). Using caesium carbonate, carbamoyl sulfones were converted into the corresponding *N*-Boc-protected imines **2** that were immediately submitted to List's organocatalytic Mannich reaction [29–30]. The resulting aldehydes **3** were not isolated (also in view of their known stereochemical lability) but directly reduced to alcohols **4** [32–33]. Purification was carried out through chromatography and, in some cases, by additional crystallization, affording these key intermediates in high ee and de (*syn* relative configuration, see Supporting Information File 1).

The *tert*-butyl urethane was then deblocked with trifluoroacetic acid. Neutralization and extraction afforded crude aminoalcohols **5a–e**, that were directly employed in the Ugi reaction. We first optimized this step using isobutyraldehyde, 5-chloro-2-thiophenecarboxylic acid and cyclohexyl isocyanide, to give the two diastereomers of compound **6a** (Table 1).

**Table 1:** Optimization of the synthesis of **6a**.

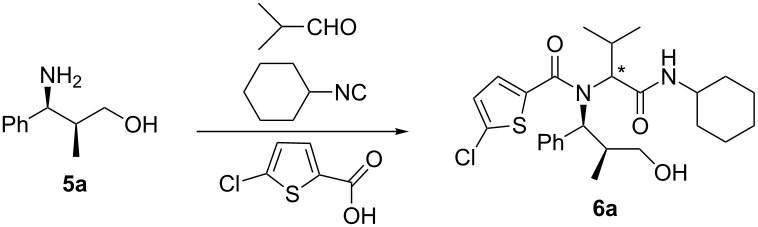						

Entry	Temp.	Time (h)	Solvent (M)	Lewis acid (equiv)	Yield^a^	dr^b^

1	25 °C	12	MeOH (0.4)	none	65%	72:28
2	25 °C	48	MeOH (0.1)	none	72%	72:28
3	25 °C	12	CF_3_CH_2_OH (0.1)	none	65%	77:23
4	0 °C	12	CF_3_CH_2_OH (0.1)	none	61%	83:17
5	−38 °C	48	THF (0.1)	ZnCl_2_·Et_2_O (1.0)	55%	89:11
6	−38 °C	48	THF (0.1)	ZnCl_2_ (1.0)	63%	73:27
7	−38 °C	48	THF (0.1)	ZnCl_2_ (1.5)	60%	80:20
8	−38 °C	48	THF (0.1)	ZnBr_2_ (1.0)	82%	91:9
9	−38 °C	48	THF (0.1)	ZnI_2_ (1.0)	71%	88:12
10	−38 °C	48	THF (0.1)	CuBr_2_ (1.0)	no react.	–
11	−38 °C	48	THF (0.1)	Cu(OTf)_2_ (1.0)	no react.	–
12	−38 °C	48	THF (0.1)	MgCl_2_ (1.0)	no react.	–
13	−38 °C	48	THF (0.1)	MgBr_2_·Et_2_O (1.0)	no react.	–
14	−38 °C	48	THF (0.1)	Yb(OTf)_3_ (0.2)	no react.	–

^a^Overall yield from aminoalcohol. ^b^Relative configuration not yet determined.

When the reaction was carried out under the classical conditions (using methanol as the solvent), only a moderate diastereoselectivity was achieved (Table 1, entry 1), which could be increased by changing the solvent to trifluoroethanol, especially effective at 0 °C. Considering the recent work by Nenajdenko et al. [17], we explored the usage of Lewis acids in an aprotic solvent in order to further improve the diastereoselectivity. We had anticipated that the binding of the Lewis acid to the free alcohol, followed by intramolecular activation of the aldehyde, would establish a cyclic transition state, thereby enabling better stereocontrol. It is indeed well-known that the Ugi reaction does not proceed in aprotic solvents such as THF at low temperature, and therefore the background, uncatalyzed reaction should not interfere. As shown in Table 1, the best results were achieved by using 1 equiv of zinc bromide (Table 1, entry 8), affording a 10:1 diastereomeric ratio and an excellent overall yield. Other zinc-based catalysts were less efficient, whereas most of the other tested Lewis acids failed to promote the reaction at all. The use of Lewis acids in methanol or trifluoroethanol afforded lower yields with no improvement of diastereoselection. It is worth noting that a 10:1 diastereoselectivity is considered excellent for isocyanide-based multicomponent reactions, due to the very low steric biases of isocyanides.

We then moved on to establish the scope of the method, varying the Boc-protected aminoalcohol, the carboxylic acid and the isocyanide (see Table 2). For a comparison, we performed all Ugi reactions either under Lewis acid-promoted conditions, or under the classical Ugi conditions (MeOH, rt). The stereochemical results were found to vary remarkably from case to case. While in some instances (products **6b**–**d**) the activation with ZnBr_2_ brought about an increase of diastereoselectivity, in other combinations of substrates, the outcome was similar (products **6h** and **6j**) or even better using the "classical" conditions (products **6e**, **6f**, **6i**). However, in all cases, the two diastereomers could be easily separated and the ratio was typically, with few exceptions, around 3:1 to 5:1. As far as the isolated yields were concerned, the Lewis acid-promoted reaction is typically less efficient, especially with aromatic isocyanides or aldehydes (compounds **6e**, **6f**, **6g**). The relative configuration of the major adduct has not yet been unambiguously determined. However, TLC, HPLC, polarimetric and NMR analogies suggest that the main diastereomer was always the same, with one notable exception: product **6f** obtained in the absence of Lewis acid. In this case, it was necessary to carry out the reaction in THF/iPrOH because the isocyanide was poorly soluble in MeOH, and thus the unexpected diastereoselectivity inversion might be due to the different solvent and not to the structure of isocyanide.

**Table 2:** Scope of the synthesis of Ugi adducts **6**.

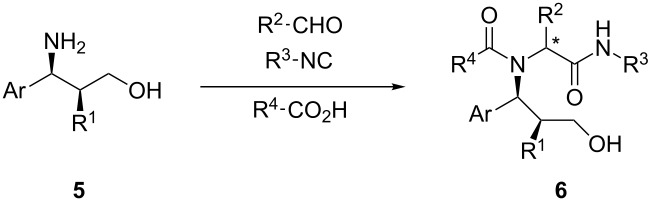								

Prod.	Ar	R^1^	R^2^	R^3^	R^4^	Cond.^a^	Yield^b^	dr

**6a**	Ph	Me	iPr	*cy*-Hex	5-Cl-2-thienyl	**A**	**82%**	**91:9**
						B	72%	72:28
**6b**	Ph	Me	iPr	*n*-C_5_H_11_	5-Cl-2-thienyl	**A**	**53%**	**85:15**
						B	82%	73:27
**6c**	2-BnOC_6_H_4_	Me	iPr	cyclohexyl	5-Cl-2-thienyl	**A**	**31%**	**82:18**
						B	40%	64:36
**6d**	Ph	Me	iPr	cyclohexyl	Et	**A**	55%	**79:21**
						B	82%	74:26
**6e**	Ph	Me	iPr	2,6-di-MeC_6_H_3_	5-Cl-2-thienyl	A	55%	65:35
						**B**	**95%**	**86:14**
**6f**	Ph	Me	iPr	4-(BnOCO)-C_6_H_4_	5-Cl-2-thienyl	A	<5%	n.d.
						**C**	**60%**	**35:65**
**6g**	Ph	Me	Ph	*n*-C_5_H_11_	5-Cl-2-thienyl	A	<20%	n.d.
						**B**	**79%**	**57:43**
**6h**	Ph	iPr	iPr	cyclohexyl	5-Cl-2-thienyl	A	40%	78:22
						**B**	**70%**	**77:23**
**6i**	2-BnOC_6_H_4_	iPr	iPr	cyclohexyl	5-Cl-2-thienyl	A	30%	64:36
						**B**	**54%**	**69:31**
**6j**	2-BnOC_6_H_4_	Bn	iPr	*t*-Bu	CbzNH-CH_2_	A	48%	80:20
						**B**	**77%**	**81:19**

^a^Overall yield from Boc aminoalcohols **4**. ^b^Relative configuration not yet determined. A: THF, −38 °C, 1 equiv of ZnBr_2_; B: MeOH, 25 °C; C: iPrOH/THF 2:1, 25 °C. All reactions carried out for 48 h at 0.1 M concentration of aminoalcohol with 1.00 equiv of aminoalcohol **5**, 1.05 equiv of aldehyde, 1.2 equiv of carboxylic acid and isocyanide and 100 mg of powdered 3 Å molecular sieves per mmol of aminoalcohol.

The synthetic route from carbamoyl sulfones **1** to peptidomimetics **6** is quite short: intermediate purification was carried out only at the level of the Boc-protected aminoalcohols **4** and of the final products **6**. Thus, this method offers an operationally simple route to enantiomerically pure complex structures like **6**, introducing up to five diversity inputs and controlling three stereogenic centers (also thanks to the final chromatography).

Compounds **6** are endowed with several functionalities that can be exploited for post-Ugi cyclization steps or as a handle for attaching further fragments: the primary alcohol and the secondary amide (which are present in all products), a protected phenol (for compounds **6c**, **6i**, **6j**), and a protected amine (**6j**). Studies towards this goal are in progress and will be reported in due course.

## Supporting Information

File 1 General remarks, experimental procedures and characterization data; ^1^H and ^13^C NMR spectra of new compounds **4** and **6** (major isomer only).
