# Supplementary material for: Diastereoselective Ugi reaction of chiral 1,3-aminoalcohols derived from an organocatalytic Mannich reaction
Source: Beilstein J Org Chem. 2016 Jan 26;12:139–43. doi: 10.3762/bjoc.12.15 (PMC4734417; doi:10.3762/bjoc.12.15)
Supplement: File 1 — General remarks, experimental procedures and characterization data; 1H and 13C NMR spectra of new compounds 4 and 6 (major isomer only). [file Beilstein_J_Org_Chem-12-139-s001.pdf]

Supporting Information

for

Diastereoselective Ugi reaction of chiral 1,3-aminoalcohols derived from organocatalytic Mannich reaction

Samantha Caputo, Andrea Basso, Lisa Moni, Renata Riva, Valeria Rocca and Luca Banfi*

Address: Department of Chemistry and Industrial Chemistry, University of Genova, I-16146 Genova, Italy

Email: Luca Banfi - banfi@chimica.unige.it
* Corresponding author

General remarks, experimental procedures and characterization data; ^{1}H and ^{13}C NMR spectra of new compounds 4 and 6 (major isomer only)

Contents

1. Experimental details and characterization of new compounds S2
2. NMR Spectra of all new compounds S12

1) Experimental details and characterization of new compounds

General remarks

NMR spectra were taken at rt in $CDCl_3$ at 300 MHz (^{1}H), and 75 MHz (^{13}C), using, as internal standard, TMS (^{1}H NMR: 0.000 ppm) or the central peak of $CDCl_3$ (^{13}C: 77.02 ppm), or the central peak of DMSO (^{1}H NMR in DMSO-d_6; 2.506 ppm), or the central peak of DMSO (^{13}C in DMSO-d_6; 39.43 ppm). Chemical shifts are reported in ppm (δ scale). Coupling constants are reported in Hertz. Peak assignments were made with the aid of gCOSY and gHSQC experiments. In ABX system, the proton A is considered upfield and B downfield. GC–MS were carried out using an HP-1 column (12 m long, 0.2 mm wide), electron impact at 70 eV, and a mass temperature of about 170 °C. Only *m*/*z* > 33 were detected. All analyses were performed (unless otherwise stated) with a constant He flow of 0.9 mL/min with initial temp. of 100 °C, init. time 2 min, rate 20 °C/min, final temp. 280 °C, inj. temp. 250 °C, det. temp. 280 °C. HRMS: samples, provided at 10 mM in DMSO, were diluted at 50 µM with acetonitrile/water 1:1, and analyzed on a UPLC Acquity system coupled to a Synapt G2 QToF mass spectrometer. MS signals were acquired from 50 to 1200 *m*/*z* both ESI positive and ESI negative ionization mode. UPLC was carried out with H_2O–CH_3CN–HCO_2H with an Acquity UPLC BEH C18, 1.7 µM, 2.1 × 50 mm column at 45 °C. IR spectra were recorded with the ATR (attenuated total reflectance) technique. TLC analyses were carried out on silica gel plates and viewed at UV (254 nm) and developed with Hanessian stain (dipping into a solution of $(NH_4)_4MoO_4{\cdot}4H_2O$ (21 g) and $Ce(SO_4)_2{\cdot}4H_2O$ (1 g) in H_2SO_4 (31 mL) and H_2O (469 mL) and warming) or, only to detect free amines after Boc deblocking, with ninhydrin (900 mg of ninhydrin in 300 mL *n*-BuOH and 9 mL AcOH, followed by warming). R_f were measured after an elution of 7–9 cm. Column chromatographies were done with the "flash" methodology using 220–400 mesh silica. Petroleum ether (40–60 °C) is abbreviated as PE. In extractive work-up, aqueous solutions were always reextracted thrice with the appropriate organic solvent. Organic extracts were always dried over Na_2SO_4 and filtered, before evaporation of the solvent under reduced pressure. All reactions using dry solvents were carried out under a nitrogen (or argon if specified) atmosphere. Petroleum ether (40–60 °C) is abbreviated as PE. In extractive work-up, aqueous solutions were always re-extracted thrice with the appropriate organic solvent. Organic extracts were always dried over Na_2SO_4 and filtered, before evaporation of the solvent under reduced pressure.

Typical procedure for the synthesis of Boc protected aminoalcohols 4 from carbamoyl sulfones 1

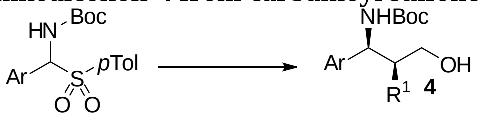

1a: Ar = Ph
1b: Ar = *o*-$BnOC_6H_4$

4a: Ar = Ph, R^1 = Me
4b: Ar = Ph, R^1 = *i*Pr
4c: Ar = *o*-$BnOC_6H_4$, R^1 = Me
4d: Ar = *o*-$BnOC_6H_4$, R^1 = *i*Pr
4e: Ar = *o*-$BnOC_6H_4$, R^1 = Bn

Carbamoyl sulfone **1a**[1] or **1b**[2] (2 mmol) was dissolved in dry THF (20 mL), treated with Cs_2CO_3 (1.303 g, 4.00 mmol) and heated at 50 °C for 3 h. After cooling, the white suspension was filtered through a celite cake, washing with THF. The filtrate was evaporated, dissolved in dry CH_3CN (20 mL), cooled to 0 °C, and treated with L-proline (19 mg, 0.4 mmol) and with the appropriate aldehyde (propionaldehyde, isovaleraldehyde or hydrocinnamaldehyde) (4 mmol). The mixture was stirred at 0 °C for 18 h, and then evaporated to dryness. The crude was taken up in water and extracted with CH_2Cl_2 (3 × 10 mL). The organic extracts were evaporated to dryness, and dissolved in MeOH (10 mL), cooled to 0 °C, and treated, in portions, with $NaBH_4$ (3 mmol). After 5 min the cooling bath was removed and the reaction stirred for 1 h at rt and then quenched with 5% aqueous NH_4H_2PO/2 M HCl 10:2. Extraction with AcOEt, evaporation and chromatography gave pure compounds **4a–e**. In the case of **4a,b** they were further crystallized.

(1*S*,2*S*)-3-(*tert*-Butoxycarbonylamino)-2-methyl-3-phenylpropanol (4a). Yield: 70%. Crystallized from Et_2O / *n*-pentane. M.p. 101-103°C (lit.[3]: 101-103°C. $[\alpha]_D$: −17.0 (c 0.5, $CHCl_3$) (lit. [3]: −15.0). *Syn*:*anti* ratio (^{1}H NMR): 97:3. E.e. was supposed to be > 99% on the basis of literature data.[1,2,4] The other spectroscopic data were coincident with those already reported.[3]

(1*S*,2*S*)-3-(*tert*-Butoxycarbonylamino)-2-(isopropyl)-3-phenylpropanol (4b). Yield from **1a**: 65%. Crystallized from Et_2O. M.p. 103-105°C (lit. [5]: 106-108°C. $[\alpha]_D$: −24.8 (c 0.5, $CHCl_3$) (lit. [5]: −26.7). *Syn*:*anti* ratio (^{1}H NMR): 97:3. E.e. was supposed to be > 96% on the basis of literature data.[2] The other spectroscopic data were coincident with those already reported.[5]

(1*S*,2*S*)-3-(2-(Benzyloxy)phenyl)-3-(*tert*-butoxycarbonylamino)-2-methylpropanol (4c). Yield from **1b**: 57%. Yellowish oil. $[\alpha]_D$: -17.7 (c 0.59, $CHCl_3$). *Syn*:*anti* ratio (^{1}H NMR): 95:5. E.e. was supposed to be > 99% on the basis of literature data.[2] ^{1}H NMR (300 MHz, $CDCl_3$, 25 °C) δ 7.50-7.30 (5 H, m, C*H* of benzyl), 7.26-7.14 (2 H, m, *H*-4, *H*-6), 7.00-6.93 (2 H, m, *H*-3, *H*-5), 6.00 (1 H, d, *J* = 9.6 Hz., N*H*), 5.17 (1 H, dd, *J* = 4.5, 9.6 Hz., C*H*NH), 5.12, 5.09 (2 H, AB system, *J* = 11.3 Hz., CH_2Ph), 3.46-3.24 (2 H, m CH_2OH), 2.15-2.02 (1 H, m, C*H*CH_3), 1.42 (9 H, s, $(CH_3)_3$C), 0.73 (3 H, d, *J* = 6.9 Hz., CH_3CH). ^{13}C NMR (75 MHz, $CDCl_3$, 25 °C) δ 156.6, 156.1 (*C*=O and *C*-2), 136.5 (quat. of benzyl), 129.0, 128.2 (*C*-4 and *C*-6), 128.9 (*C*-1), 128.7, 127.5 (ortho and meta *C* of benzyl), 128.2 (para *C* of benzyl), 121.1 (*C*-5), 112.3 (*C*-3), 79.5 (*C*$(CH_3)_3$), 70.3 (PhCH_2O), 65.1 (CH_2OH), 53.0 (*C*HNH), 41.8 (*C*HCH_3), 28.4 (C$(CH_3)_3$), 11.3 (CHCH_3). I.r. (ATR): ν_{max} 3436, 2973, 2931, 1687, 1601, 1490, 1452, 1391, 1365, 1287, 1237, 1163, 1092, 1075, 1035, 1005, 882, 751, 696, 624 cm^{-1}. HRMS (ESI+). Calcd for $C_{22}H_{30}NO_4$ (M + H^+) 372.2175; Found 372.2171.

(*1S*,*2S*)-3-(2-(Benzyloxy)phenyl)-3-(*tert*-butoxycarbonylamino)-2-isopropylpropanol (4d). Yield from **1b**: 39%. Colourless oil. $[\alpha]_D$: -44.2 (c 0.5, $CHCl_3$) *Syn*:*anti* ratio (^{1}H NMR) > 96%. E.e. was supposed to be > 96% on the basis of literature data.[2] ^{1}H NMR (300 MHz, $CDCl_3$, 25 °C) δ 7.48-7.30 (5 H, m, C*H* of benzyl), 7.27-7.18 (2 H, m, *H*-4, *H*-6), 6.98-6.91 (2 H, m, *H*-3, *H*-5), 5.86 (1 H, d, *J* = 9.6 Hz., N*H*), 5.11 (2 H, s, CH_2Ph), 5.05 (1 H, dd, *J* = 9.0, 9.6 Hz., C*H*NH), 3.52-3.42 (2 H, m CH_2OH), 2.15-1.80 (2 H, m, C*H*CH_3, C*H*$(CH_3)_2$), 1.41 (9 H, s, $(CH_3)_3$C), 0.92 (3 H, d, *J* = 6.6 Hz., CH_3), 0.83 (3 H, d, *J* = 6.6 Hz., CH_3). ^{13}C NMR (75 MHz, $CDCl_3$, 25 °C) δ 156.3, 155.7 (*C*=O and *C*-2), 136.4 (quat. of benzyl), 129.6 (*C*-1), 129.4, 128.3 (*C*-4 and *C*-6), 128.6, 127.6 (ortho and meta *C* of benzyl), 128.1 (para *C* of benzyl), 121.2 (*C*-5), 112.4 (*C*-3), 79.1 (*C*$(CH_3)_3$), 70.5 (PhCH_2O), 60.6 (CH_2OH), 53.5 (*C*HNH), 50.9 (*C*H*i*Pr), 28.3 (C$(CH_3)_3$), 26.3 (*C*HMe_2), 22.3, 18.0 (CH$(CH_3)_2$). I.r. (ATR): ν_{max} 3442, 2959, 1692, 1601, 1587, 1490, 1452, 1390, 1365, 1286, 1235, 1165, 1097, 1040, 1012, 912, 884, 856, 799, 751, 733, 696, 624 cm^{-1}. HRMS (ESI+). Calcd for $C_{24}H_{34}NO_4$ (M + H^+) 400.2488; Found 400.2492.

(*1S*,*2S*)-2-Benzyl-3-(2-(benzyloxy)phenyl)-3-(*tert*-butoxycarbonylamino)propanol (4e). Yield from **1b**: 76%. Colourless oil. $[\alpha]_D$: -13.9 (c 1, $CHCl_3$). *Syn*:*anti* ratio (^{1}H NMR): > 95:5. E.e. was demonstrated to be > 95% by Mosher's ester analysis. ^{1}H NMR (300 MHz, $CDCl_3$, 25 °C) δ. 7.50-7.33 (5 H, m), 7.32-7.10 (5 H, m), 7.03-6.93 (4 H, m), 6.05 (1 H, d, *J* = 9.6 Hz., N*H*), 5.21 (1 H, dd, *J* = 9.6, 6.0 Hz., C*H*NHBoc), 5.14-5.10 (2 H, AB syst., *J* = 11.4 Hz., OCH_2Ph), 3.30 (1 H, t, *J* = 5.3 Hz., CH_2OH), 2.92 (1 H, t, *J* = 6.5 Hz., O*H*), 2.79 (1 H, d, *J* = 10.2 Hz., C*H*HPh), 2.38 (1 H, t, *J* = 12.3 Hz., C*H*HPh), 2.34-2.20 (1 H, m, C*H*-CH_2Ph), 1.43 (9 H, s, $(CH_3)_3$C). ^{13}C NMR (75 MHz, $CDCl_3$, 25 °C) δ 156.4, 156.0 (*C*=O and *C*–OBn), 140.4, 136.3, 129.3 (other aromatic quat.), 129.3, 128.8 (x 2), 128.7 (x 2), 128.4, 128.2 (x 3), 127.6 (x 2), 125.4, 121.3, 112.3, 79.5 (O*C*$(CH_3)_3$), 70.4 (OCH_2Ph), 61.7 (CH_2OH), 53.5 (*C*HNH), 48.2 (*C*H–CH_2Ph), 32.7 (C–CH_2Ph), 28.3 (C$(CH_3)_3$). I.r. (ATR): ν_{max} 3437, 3030, 2977, 2931, 1688, 1602, 1587, 1490, 1452, 1391, 1365, 1283, 1236, 1162, 1120, 1089, 1027, 1009, 916, 878, 750, 737, 697, 624 cm^{-1}. HRMS (ESI+). Calcd for $C_{28}H_{34}NO_4$ (M + H^+) 448.2488; Found 448.2490.

5-Chloro-*N*-((*R* or *S*)-1-(cyclohexylamino)-3-methyl-1-oxobutan-2-yl)-*N*-((1*S*,2*S*)-3-hydroxy-2-methyl-1-phenylpropyl)thiophene-2-carboxamide (6a).

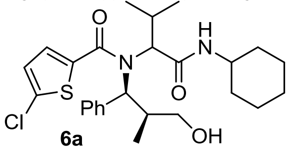

6a

Method A. A solution of Boc aminoalcohol **4a** (133 mg, 0.50 mmol) in dry CH_2Cl_2 (9 mL), was cooled to 0 °C, and treated with trifluoroacetic acid (4.2 mL). After stirring for 1 h at 0 °C, the solution was evaporated to dryness, and taken up with 1 M aqueous NaOH (15 mL) and AcOEt (15 mL). The phases were separated and the aqueos one re-extracted twice with AcOEt. The organic extracts were washed with saturated queous NaCl, dried, and evaporated to dryness to give crude **5a** as an oil. It was taken up in dry THF (2 mL), cooled to −38 °C, and treated, in this order, with isobutyraldehyde (48 µL, 0.53 mmol), 3 Å powdered molecular sieves (50 mg), zinc dibromide (113 mg, 0.50 mmol) previously dissolved in dry THF (3 mL), 5-chloro-2-thiophenecarboxylic acid (98 mg, 0.60 mmol), and cyclohexyl isocyanide (75 µL, 0.60 mmol). The mixture was stirred for 48 h at −38 °C. Then it was evaporated to dryness and chromatographed (PE/AcOEt 40:60 + 1% MeOH) to give first the minor diastereomer (R_f = 0.49) (18 mg, 7%), and then the major one (R_f = 0.32) (183 mg, 75%). Overall yield (from **4a**): 82%. The diastereomeric ratio was also determined by HPLC on the crude product and resulted = 91:9. HPLC conditions: Column LUNA C8 150 × 4.6 mm. Injected volume: 5µL (1 mg/mL). Flow: 0.75 mL/min. Temp = 22 °C. Isocratic elution: H_2O/CH_3CN 35:65. Detection: UV (220 µM). t_R: 12.7 min (major), 14.4 min (minor).

Method B. Crude **5a** was prepared as above. Then it was taken up in dry MeOH (5 mL), and treated, at rt, with isobutyraldehyde (48 µL, 0.53 mmol), 3 Å powdered molecular sieves (50 mg), 5-chloro-2-thiophenecarboxylic acid (98 mg, 0.60 mmol), and cyclohexyl isocyanide (75 µL, 0.60 mmol). The mixture was stirred for 48 h at rt. Then it was evaporated to dryness and chromatographed (PE/AcOEt 40:60 + 1% MeOH) to give first the minor diastereomer (R_f = 0.49) (49 mg, 20%), and then the major one (R_f = 0.32) (127 mg, 52%). Overall yield (from **4a**): 72%. The diastereomeric ratio was also determined by HPLC on the crude product and resulted = 72:28.

Major diastereomer: colourless oil. $[\alpha]_D$: +120.9 (c 0.575, $CHCl_3$). ^{1}H NMR (300 MHz, d6-DMSO, 90 °C): δ 7.60 (1 H, d, *J* = 4.0 Hz., *H*-3 of thienyl), 7.40-7.20 (6 H, m, C*H* of phenyl and N*H*), 7.13 (1 H, d, *J* = 4.0 Hz., *H*-4 of thienyl), 5.03 (1 H, d, *J* = 10.8 Hz., C*H*-Ph), 4.30-4.00 (1 H, broad m, O*H*), 3.63 (1 H, d, *J* = 9.6 Hz., C*H*-C=O), 3.32 (1 H, dd, *J* = 10.5, 2.3 Hz., C*H*H-OH), 3.28-3.14 (1 H, m, C*H*NH), 3.05 (1 H, dd, *J* = 10.5, 5.2 Hz., C*H*H-OH), 2.82-2.66 (1 H, m, C*H*(Me)-CH_2OH), 2.49-2.34 (1 H m, C*H*$(CH_3)_2$), 1.80-0.80 (10 H, m, cyclohexyl), 1.22 (3 H, d, *J* = 6.6 Hz., C*H*$_3$CH), 0.92 (3 H, d, *J* = 6.6 Hz., C*H*$_3$CHCH$_3$), 0.88 (3 H, d, *J* = 6.6 Hz., C*H*$_3$CHCH$_3$). ^{13}C NMR (75 MHz, d6-DMSO, 90 °C): δ 168.2, 164.6 (*C*=O), 138.3, 138.0, 131.7 (aromatic quat.), 129.2 (*C*-3 of thienyl), 128.6 (x 2), 127.2 (x 2), 126.8 (*C*H of phenyl), 126.4 (*C*-4 of thienyl), 68.9 (*C*H-C=O), 65.9 (*C*H-Ph), 62.7 (*C*H$_2$OH), 46.6 (*C*HNH), 35.0 (*C*HCH$_3$), 27.8 (*C*H(CH$_3$)$_2$, 31.2, 31.0, 24.7, 23.4, 23.3 (*C*H$_2$ of cyclohexyl), 20.2, 19.4 ((*C*H$_3$)$_2$CH), 14.6 (*C*H$_3$CH). I.r. (ATR): ν_{max} = 3385, 3285, 2970, 2930, 2854, 1725, 1646, 1601, 1544, 1451, 1426, 1389, 1370, 1327, 1301, 1277, 1076, 1044, 1004, 804, 786, 757, 729, 699, 673, 662, 633. cm^{-1}. HRMS (ESI+). Calcd for $C_{26}H_{36}ClN_2O_3S$ (M + H^+) 491.2135; Found 491.2144.

Minor diastereomer: colourless oil. $[\alpha]_D$: −71.9 (c 0.51, $CHCl_3$). ^{1}H NMR (300 MHz, d6-DMSO, 120 °C): δ 7.93 (1 H, broad s, N*H*), 7.53 (1 H, d, *J* = 4.0 Hz., *H*-3 of thienyl), 7.45-7.30 (5 H, m, C*H* of phenyl), 7.15 (1 H, d, *J* = 4.0 Hz., *H*-4 of thienyl), 5.25 (1 H, d, *J* = 11.1 Hz., C*H*-Ph), 3.92 (1 H, broad t, O*H*), 3.77-3.65 (1 H, m, C*H*NH), 3.61 (d, *J* = 10.8 Hz., C*H*-C=O), 3.27 (1 H, d, *J* = 10.5 Hz., C*H*H-OH), 3.04 (1 H, dd, *J* = 10.5, 5.4 Hz., C*H*H-OH), 2.80-2.63 (1 H, m, C*H*(Me)-CH_2OH), 2.60-2.46 (1 H m, C*H*$(CH_3)_2$), 1.85-0.80 (10 H, m, cyclohexyl), 1.03 (3 H, d, *J* = 6.6 Hz., C*H*$_3$CH), 0.71 (3 H, d, *J* = 6.6 Hz., C*H*$_3$CHCH$_3$), 0.10 (3 H, d, *J* = 6.6 Hz., C*H*$_3$CHCH$_3$).

Important note: the relative configuration of major and minor diastereomers was not determined.

5-Chloro-*N*-((*R* or *S*)- 3-methyl-1-oxo-1-(pentylamino)butan-2-yl)-*N*-((1*S*,2*S*)-3-hydroxy-2-methyl-1-phenylpropyl)thiophene-2-carboxamide (6b).

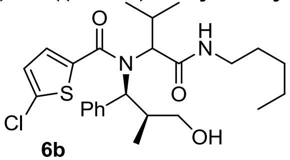

6b

It was prepared following the same procedures (A and B) used for **6a**. Overall yield: 53% (method A) and 82% (method B). The diastereomeric ratio was determined by HPLC on the crude product and resulted = 85:15 (method A) or 73:27 (method B). HPLC conditions: Column Phenyl C6 150 × 3 mm. Injected volume: 5 µL (1 mg/mL). Flow: 0.34 mL/min. Temp = 31 °C. Gradient elution: from H_2O/CH_3CN 90:10 to H_2O/CH_3CN 0:100 in 20 min. Detection: UV (220 µM). t_R: 18.8 min (major), 19.3 min (minor).

Major diastereomer: colourless oil. R_f = 0.44 (AcOEt/PE 3:2 + 1% MeOH). $[\alpha]_D$: +120.7 (c 0.525, $CHCl_3$). ^{1}H NMR (300 MHz, d6-DMSO, 90 °C): δ 7.59 (1 H, d, *J* = 4.0 Hz., *H*-3 of thienyl), 7.42-7.20 (6 H, m, C*H* of phenyl and N*H*), 7.12 (1 H, d, *J* = 4.0 Hz., *H*-4 of thienyl), 4.94 (1 H, d, *J* = 10.7 Hz., C*H*-Ph), 4.30-3.90 (1 H, broad m, O*H*), 3.70 (1 H, d, *J* = 9.7 Hz., C*H*-C=O), 3.31 (1 H, dd, *J* = 10.4, 2.8 Hz., C*H*H-OH), 3.04 (1 H, dd, *J* = 10.5, 5.4 Hz., C*H*H-OH), 2.84-2.60 (3 H, m, CH_2NH, C*H*(Me)-CH_2OH), 2.38 (1 H, d of heptuplets, J_d = 9.6, J_h = 6.6 Hz., C*H*(CH_3)$_2$), 1.30-1.08 (6 H, m, CH_2–CH_2–C$H_2$$CH_3$), 1.20 (3 H, d, *J* = 6.6 Hz., CH_3CH), 0.90 (3 H, d, *J* = 6.6 Hz., CH_3CHCH_3), 0.86 (3 H, d, *J* = 6.6 Hz., CH_3CHCH_3), 0.86 (3 H, t, *J* = 7.0 Hz., C$H_3$$CH_2$). ^{13}C NMR (75 MHz, d6-DMSO, 90 °C): δ 168.8, 164.6 (*C*=O), 138.4, 138.3, 131.6 (aromatic quat.), 129.1 (*C*-3 of thienyl), 128.6 (x 2), 127.1 (x 2), 126.8 (*C*H of phenyl), 126.3 (*C*-4 of thienyl), 68.8 (*C*H-C=O), 65.6 (*C*H-Ph), 62.8 (*C*H_2OH), 38.0 (*C*H_2NH), 35.5 (*C*HCH_3), 28.0, 27.6, 21.1 (*C*H_2*C*H_2*C*H_2CH), 27.6 (*C*H(CH_3)$_2$, 20.0, 19.4 ((*C*H_3)$_2$CH), 14.7 (*C*H_3CH), 13.0 (*C*$H_3$$CH_2$). I.r. (ATR): ν_{max} = 3401, 2960, 1655, 1049, 1024, 998, 824, 760, 729, 701 cm^{-1}. HRMS (ESI+). Calcd for $C_{25}H_{36}ClN_2O_3S$ (M + H$^+$) 479.2135; Found 479.2145.

Minor diastereomer: colourless oil. R_f = 0.68 (AcOEt/PE 3:2 + 1% MeOH). Important note: the relative configuration of major and minor diastereomers was not determined.

5-Chloro-*N*-((*R* or *S*)-1-(cyclohexylamino)-3-methyl-1-oxobutan-2-yl)-*N*-((1*S*,2*S*)-1-((2-benzyloxy)phenyl)-3-hydroxy-2-methylpropyl)thiophene-2-carboxamide (6c).

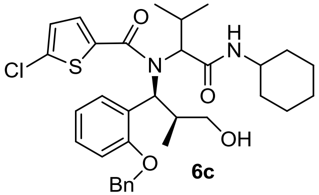

6c

It was prepared following the same procedures (A and B) used for **6a**. Overall yield: 31% (method A) and 40% (method B). The diastereomeric ratio was determined by HPLC on the crude product and resulted = 82:18 (method A) or 64:36 (method B). HPLC conditions: Column Phenyl C6 150 × 3 mm. Injected volume: 5 µL (1 mg/mL). Flow: 0.34 mL/min. Temp = 23 °C. Gradient elution: from H_2O/CH_3CN 90:10 to H_2O/CH_3CN 0:100 in 20 min. Detection: UV (220 µM). t_R: 20.6 min (major), 21.6 min (minor).

Major diastereomer: colourless oil. R_f = 0.22 (AcOEt/PE 3:2 + 1% MeOH). $[\alpha]_D$: +116.7 (c 0.47, $CHCl_3$). ^{1}H NMR (300 MHz, d6-DMSO, 90 °C): δ 7.62 (1 H, d, *J* = 4.1 Hz., *H*-3 of thienyl), 7.48 (1 H, d, *J* = 6.9 Hz., *H*-6 of aryl), 7.34-7.23 (5 H, m, C*H* of benzyl), 7.20 (1 H, dt, J_t = 7.7, J_d = 1.5 Hz., *H*-4 of aryl), 7.10 (1 H, d, *J* = 7.2 Hz., N*H*), 6.96 (1 H, d, *J* = 7.8 Hz., *H*-3 of aryl), 6.91 (1 H, d, *J* = 4.1 Hz., *H*-4 of thienyl), 6.88 (1 H, t, *J* = 7.5 Hz., *H*-5 of aryl), 5.81 (1 H, d, *J* = 11.4

Hz., C*H*-Ar), 5.01 and 4.90 (2 H, AB syst., *J* = 12.3 Hz., CH_2Ph), 4.26 (1 H, broad t, O*H*), 3.52 (1 H, d, *J* = 8.3 Hz., C*H*-C=O), 3.38 (1 H, dt, J_d= 10.5, J_t =3.5 Hz., C*H*H-OH), 3.23-3.05 (2 H, m, CH*H*-OH, C*H*NH), 2.80-2.65 (1 H, m, C*H*(Me)-CH_2OH), 2.48 (1 H, octuplet, *J* = 6.6 Hz., C*H*(CH_3)$_2$), 1.65-0.70 (10 H, m, CH_2 of cyclohexyl), 1.26 (3 H, d, *J* = 6.9 Hz., CH_3CH), 0.96 (3 H, d, *J* = 6.6 Hz., CH_3CHCH_3), 0.94 (3 H, d, *J* = 6.6 Hz., CH_3CHCH_3). ^{13}C NMR (75 MHz, d6-DMSO, 90 °C): δ 168.5, 164.4 (*C*=O), 156.5 (*C*-2 of aryl), 139.3, 136.3, 132.1, 125.1 (aromatic quat.), 129.4 (*C*-6 of aryl), 129.1 (*C*-3 of thienyl), 128.3 (*C*-4 of aryl), 127.6 (x 2), 126.9, 126.8 (x 2) (aromatic *C*H of benzyl), 126.3 (*C*-4 of thienyl), 119.7 (*C*-5 of Ar), 112.0 (*C*-3 of aryl), 69.5 (*C*H_2Ph), 68.7 (*C*H-C=O), 62.6 (*C*H_2OH), 57.9 (*C*H-Ar), 46.2 (*C*HNH), 34.3 (*C*HCH_3), 28.3 (*C*H(CH_3)$_2$, 31.3, 31.0, 24.6, 23.22, 23.17 (*C*H_2 of cyclohexyl), 21.2, 19.7 ((*C*H_3)$_2$CH), 14.3 (*C*H_3CH). I.r. (ATR): ν_{max} = 3400, 3282, 2970, 2928, 2853, 1739, 1647, 1427, 1010, 749, 695. cm^{-1}. HRMS (ESI+). Calcd for $C_{33}H_{42}ClN_2O_4S$ (M + H$^+$) 597.2554; Found 597.2557.

Minor diastereomer: colourless oil. R_f = 0.40 (AcOEt/PE 3:2 + 1% MeOH). Important note: the relative configuration of major and minor diastereomers was not determined.

N-((R or S)-1-(cyclohexylamino)-3-methyl-1-oxobutan-2-yl)-N-((1S,2S)-1-phenyl-3-hydroxy-2-methylpropyl)propionamide (6d).

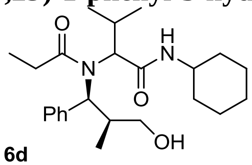

6d

It was prepared following the same procedures (A and B) used for **6a**. Overall yield: 55% (method A) and 82% (method B). The diastereomeric ratio was determined by HPLC on the crude product and resulted = 79:21 (method A) or 74:26 (method B). HPLC conditions: Column Phenyl C6 150 × 3 mm. Injected volume: 5 μL (0.5 mg/mL). Flow: 0.34 mL/min. Temp = 25 °C. Gradient elution: from H_2O/CH_3CN 90:10 to H_2O/CH_3CN 0:100 in 20 min. Detection: UV (220 μM). t_R: 15.7 min (major), 16.9 min (minor).

Major diastereomer: colourless oil. R_f = 0.23 (AcOEt/PE 1:1 + 1% MeOH). $[\alpha]_D$: +96.1 (c 0.485, $CHCl_3$). ^{1}H NMR (300 MHz, $CDCl_3$, 28 °C): δ 7.59 (1 H, broad d, *J* = 6.5 Hz., N*H*), 7.28 (5 H, s, C*H* of phenyl), 4.87 (1 H, d, *J* = 11.0 Hz., C*H*-Ph), 3.61 (1 H, d, *J* = 9.9 Hz., C*H*H-OH), 3.42 (1 H, dd, *J* = 10.3, 3.4 Hz., CH*H*-OH), 3.32-3.15 (2 H, m, C*H*-C=O, C*H*NH), 2.87-2.74 (1 H, m, C*H*(Me)-CH_2OH), 2.68 (2 H, q, *J* = 7.1 Hz., C$H_2$$CH_3$), 2.60-2.43 (1 H, broad m, C*H*(CH_3)$_2$), 1.70-0.68 (10 H, m, CH_2 of cyclohexyl), 1.26 (3 H, d, *J* = 6.9 Hz., CH_3CH), 1.23 (3 H, t, *J* = 7.1 Hz., CH_2CH_3), 0.96 (3 H, d, *J* = 6.6 Hz., CH_3CHCH_3), 0.93 (3 H, d, *J* = 6.6 Hz., CH_3CHCH_3). ^{13}C NMR (75 MHz, $CDCl_3$, 28 °C): δ 176.8, 171.1 (*C*=O), 136.6 (aromatic quat.), 129.3 (x 2), 128.5 (x 2), 128.3 (aromatic *C*H), 71.0 (*C*H-C=O), 64.7 (*C*H_2OH, *C*H-Ar), 46.8 (*C*HNH), 35.1 (*C*HCH_3), 32.3, 32.0, 25.6, 24.2, 24.1 (*C*H_2 of cyclohexyl), 28.9 (*C*H(CH_3)$_2$, 28.7 (*C*$H_2$$CH_3$), 22.1, 20.4 ((*C*$H_3$)$_2$CH), 15.2 (*C*$H_3$CH), 10.0 (*C*$H_3$$CH_2$). I.r. (ATR): ν_{max} = 3287, 2970, 2930, 2855, 1739, 1652, 1542, 1451, 1370, 1259, 1228, 1217, 1026, 1010, 988, 798, 755, 700, 621 cm^{-1}. HRMS (ESI+). Calcd for $C_{24}H_{39}CN_2O_3$ (M + H$^+$) 403.2961; Found 403.2979.

Minor diastereomer: colourless oil. R_f = 0.29 (AcOEt/PE 1:1 + 1% MeOH). Important note: the relative configuration of major and minor diastereomers was not determined.

5-Chloro-N-((R or S)-1-(2,6-dimethylphenyl)-3-methyl-1-oxobutan-2-yl)-N-((1S,2S)-3-hydroxy-2-methyl-1-phenylpropyl)thiophene-2-carboxamide (6e).

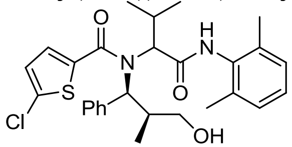

6e

It was prepared following the same procedures (A and B) used for **6a**. Overall yield: 55% (method A) and 95% (method B). The diastereomeric ratio was determined by HPLC on the crude product and resulted = 65:35 (method A) or 86:14 (method B). HPLC conditions: Column Phenyl C6 150 × 3 mm. Injected volume: 5 µL (1 mg/mL). Flow: 0.34 mL/min. Temp = 25 °C. Gradient elution: from H_2O/CH_3CN 90:10 to H_2O/CH_3CN 0:100 in 20 min. Detection: UV (220 µM). t_R: 19.4 min (major), 19.9 min (minor).

Major diastereomer: colourless oil. R_f = 0.44 (AcOEt/PE 3:2 + 1% MeOH). $[\alpha]_D$: +91.8 (c 0.52, $CHCl_3$). ^1H NMR (300 MHz, d6-DMSO, 90 °C): δ 8.90 (1 H, s, N*H*), 7.65 (1 H, d, *J* = 4.0 Hz., *H*-3 of thienyl), 7.51-7.45 (2 H, m, *H*-3, *H*-5 of dimethylphenyl), 7.30-7.22 (3 H, m), 7.15 (1 H, d, *J* = 4.0 Hz., *H*-4 of thienyl), 7.10-6.93 (3 H, m), 5.05 (1 H, d, *J* = 10.5 Hz., C*H*-Ph), 4.15 (1 H, broad t, O*H*), 3.96 (1 H, d, *J* = 9.3 Hz., C*H*-C=O), 3.29 (1 H, dt, J_d = 10.4, J_t = 3.4 Hz., C*H*H-OH), 2.99 (1 H, dd, *J* = 10.4, 5.4 Hz., C*H*H-OH), 2.84-2.67 (1 H, m, C*H*(Me)-CH_2OH), 2.66-2.53 (1 H, m, C*H*$(CH_3)_2$), 1.89 (6 H, s, C*H*$_3$Ar), 1.22 (3 H, d, *J* = 6.6 Hz., C*H*$_3$CH), 1.12 (3 H, d, *J* = 6.6 Hz., C*H*$_3$CHCH_3), 0.96 (3 H, d, *J* = 6.6 Hz., C*H*$_3$CHCH_3). ^{13}C NMR (75 MHz, d6-DMSO, 90 °C): δ 167.3, 164.6 (*C*=O), 138.4, 138.0, 134.3 (x 2), 134.0, 131.6 (aromatic quat.), 129.0 (*C*-3 of thienyl), 129.1 (x 2), 127.4 (x 2), 127.1, 127.0 (x 2), 125.7 (other aromatic *C*H), 126.4 (*C*-4 of thienyl), 69.0 (*C*H-C=O), 66.6 (*C*H-Ph), 62.7 (*C*H_2OH), 36.3 (*C*HCH_3), 28.6 (*C*H$(CH_3)_2$), 20.3, 19.7 ((*C*$H_3)_2$CH), 17.6 (*C*H_3Ar), 15.0 (*C*H_3CH). I.r. (ATR): ν_{max} = 3418, 3236, 2970, 1663, 1592, 1522, 1469, 1429, 1389, 1371, 1328, 1300, 1261, 1088, 1032, 1004, 801, 767, 699, 677, 661, 640 cm^{-1}. HRMS (ESI+). Calcd for $C_{25}H_{36}ClN_2O_3S$ ($M + H^+$) 513.1979; Found 513.1989.

Minor diastereomer: colourless oil. R_f = 0.79 (AcOEt/PE 3:2 + 1% MeOH). Important note: the relative configuration of major and minor diastereomers was not determined.

***N*-((*R* or *S*)-1-((4-benzyloxycarbonylphenyl)amino)-3-methyl-1-oxobutan-2-yl)-5-Chloro-*N*-((1*S*,2*S*)-3-hydroxy-2-methyl-1-phenylpropyl)thiophene-2-carboxamide (6f).**

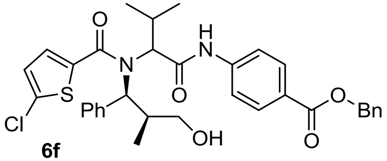

6f

Method C. A solution of Boc aminoalcohol **4a** (133 mg, 0.50 mmol) in dry CH_2Cl_2 (9 mL), was cooled to 0 °C, and treated with trifluoroacetic acid (4.2 mL). After stirring for 1 h at 0 °C, the solution was evaporated to dryness, and taken up with 1 M aqueous NaOH (15 mL) and AcOEt (15 mL). The phases were separated and the aqueos one re-extracted twice with AcOEt. The organic extracts were washed with saturated queous NaCl, dried, and evaporated to dryness to give crude **5a** as an oil. It was taken up in dry THF (1.65 mL) and dry iPrOH (3.35 mL) and treated, in this order, with isobutyraldehyde (48 µL, 0.53 mmol), 3 Å powdered molecular sieves (50 mg), 5-chloro-2-thiophenecarboxylic acid (98 mg, 0.60 mmol), and benzyl 4-isocyanobenzoate[6] (142 mg, 0.60 mmol). The mixture was stirred for 48 h at rt. Then it was evaporated to dryness and chromatographed (PE/AcOEt 2:1 + 1% MeOH) to give first the major diastereomer (R_f = 0.47) (171 mg, 55.0%), and then the minor one (R_f = 0.23) (92 mg, 29.8%). Overall yield (from **4a**): 60%. The diastereomeric ratio was also determined by HPLC on the crude product and resulted = 65:35. HPLC conditions: Column LUNA C8 150 × 4.6 mm. Injected volume: 5 µL (1 mg/mL). Flow: 0.75 mL/min. Temp = 22 °C. Isocratic elution: H_2O/CH_3CN 35:65. Detection: UV (220 µM). t_R: 12.7 min (major), 14.4 min (minor). HPLC conditions: Column Phenyl C6 150 × 3 mm. Injected volume: 5 µL (0.5 mg/mL). Flow: 0.34 mL/min. Temp = 23 °C. Gradient elution: from H_2O/CH_3CN 90:10 to H_2O/CH_3CN 0:100 in 20 min. Detection: UV (220 µM). t_R: 21.4 min (minor), 20.9 min (major).

Major diastereomer: colourless oil. $[\alpha]_D$: –137.0 (c 0.50, $CHCl_3$). ^1H NMR (300 MHz, d6-DMSO, 90 °C): δ 10.50 (1 H, broad s, N*H*), 8.02 (2 H, d, *J* = 8.7 Hz., *H* ortho to CO_2Bn), 7.72 (2 H, d, *J* = 8.7 Hz., *H* meta to CO_2Bn), 7.58 (2 H, d, *J* = 7.2 Hz.), 7.50-7.30 (9 H, m), 7.21 (1 H, d, *J* = 4.0 Hz., *H*-4 of thienyl), 5.37 (2 H, s, OC*H*$_2$Ph), 5.34 (1 H, d, *J* = 10.8 Hz., C*H*-Ph), 4.13 (1 H, t, *J* = 4.6 Hz., O*H*), 3.90-3.75 (1 H, broad m, C*H*-C=O), 3.32-3.22 (1 H, m, C*H*H-OH), 3.12-3.00 (1 H, m, C*H*H-OH), 2.80-2.60 (2 H, m, C*H*(Me)-CH_2OH, C*H*$(CH_3)_2$), 1.97 (3 H, d, *J* = 6.6 Hz., C*H*$_3$CH), 0.77 (3 H, d, *J* = 6.6 Hz., C*H*$_3$CHCH_3), 0.17 (3 H, d, *J* =

6.6 Hz., C*H*$_3$CHCH$_3$). ^{13}C NMR (75 MHz, d6-DMSO, 90 °C): δ 168.9, 164.7, 164.6 (*C*=O), 141.9, 136.7, 135.8, 127.7, 126.6, 124.6 (aromatic quat.), 129.9 (*C* ortho to CO_2Bn), 129.2 (x 2), 127.9 (x 3), 127.6, 127.4 (x 2), 127.2 (x 2), 126.74, 126.67 (other aromatic *C*H), 119.0 (*C* meta to CO_2Bn), 69.3 (*C*H-C=O), 65.4 (*C*H$_2$Ph), 64.7 (*C*HPh), 62.3 (*C*H$_2$OH), 35.8 (*C*HCH$_3$), 26.4 (*C*H(CH$_3$)$_2$), 18.8, 17.9 ((*C*H$_3$)$_2$CH), 13.6 (*C*H$_3$CH). I.r. (ATR): ν_{max} = 2964, 1715, 1688, 1585, 1533, 1436, 1410, 1259, 1212, 1173, 1095, 1014, 855, 792, 769, 752, 737, 695, 661, 635 cm^{-1}. HRMS (ESI+). Calcd for $C_{34}H_{36}ClN_2O_5S$ (M + H$^+$) 619.2033; Found 619.2038.

Minor diastereomer: colourless oil. $[\alpha]_D$: +114.0 (c 0.61, $CHCl_3$). ^{1}H NMR (300 MHz, d6-DMSO, 90 °C): δ 9.68 (1 H, s, N*H*), 7.86 (2 H, d, *J* = 8.7 Hz., *H* ortho to CO_2Bn), 7.61 (1 H, d, *J* = 4.0 Hz., *H*-3 of thienyl), 7.48-7.30 (7 H, m), 7.23 (2 H, d, *J* = 8.7 Hz., *H* meta to CO_2Bn), 7.18 (1 H, d, *J* = 4.0 Hz., *H*-4 of thienyl), 7.14 (2 H, dt, J_t = 7.5, J_d = 0.9 Hz.), 7.04 (1 H, dt, J_t = 7.2, J_d = 1.5 Hz.), 5.34 (2 H, s, OC*H*$_2$Ph), 5.17 (1 H, d, *J* = 10.8 Hz., C*H*-Ph), 4.23 (1 H, broad t, O*H*), 3.87 (1 H, d, *J* = 9.6 Hz., C*H*-C=O), 3.36 (1 H, dt, J_d = 10.1, J_t = 3.0 Hz., C*H*H-OH), 3.12 (1 H, dt, J_d = 10.1, J_t = 5.0 Hz., CH*H*-OH), 2.85-2.70 (1 H, m, C*H*(Me)-CH$_2$OH), 2.58 (1 H, d of heptuplets, J_d = 9.6, J_t = 6.6 Hz., C*H*(CH$_3$)$_2$), 1.25 (3 H, d, *J* = 6.6 Hz., C*H*$_3$CH), 0.98 (3 H, d, *J* = 6.6 Hz., C*H*$_3$CHCH$_3$), 0.97 (3 H, d, *J* = 6.6 Hz., C*H*$_3$CHCH$_3$). ^{13}C NMR (75 MHz, d6-DMSO, 90 °C): δ 168.2, 165.1, 164.6 (*C*=O), 141.9, 137.8, 137.4, 135.8, 132.2, 124.0 (aromatic quat.), 129.6 (*C*-3 of thienyl), 129.3 (*C* ortho to CO_2Bn), 128.4 (x 2), 127.09 (x 2), 127.4, 127.3 (x 2), 127.2 (x 2), 127.0 (other aromatic *C*H), 126.6 (*C*-4 of thienyl), 118.7 (*C* meta to CO_2Bn), 69.0 (*C*H-C=O), 65.42 (*C*H-Ph), 65.36 (*C*H$_2$Ph), 62.6 (*C*H$_2$OH), 34.7 (*C*HCH$_3$), 27.7 (*C*H(CH$_3$)$_2$), 20.2, 19.3 ((*C*H$_3$)$_2$CH), 14.5 (*C*H$_3$CH). I.r. (ATR): ν_{max} = 2964, 1715, 1688, 1585, 1533, 1436, 1410, 1259, 1212, 1173, 1095, 1014, 855, 792, 769, 752, 737, 695, 661, 635 cm^{-1}. HRMS (ESI+). Calcd for $C_{34}H_{36}ClN_2O_5S$ (M + H$^+$) 619.2033; Found 619.2041.

Important note: the relative configuration of major and minor diastereomers was not determined. However, from tlc, HPLC, $[\alpha]_D$ and NMR analogies, the major diastereomer of **6f** most likely corresponds to the minor ones in all other cases.

5-Chloro-*N*-((*R* or *S*)-2-oxo-2-(pentylamino)-1-phenyletan-1-yl)-*N*-((1*S*,2*S*)-3-hydroxy-2-methyl-1-phenylpropyl)thiophene-2-carboxamide (6g).

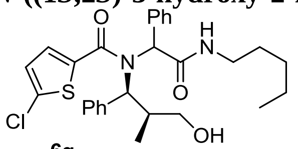

6g

It was prepared following the same procedure (B) used for **6a**. Overall yield: 79%. The diastereomeric ratio was determined by HPLC on the crude product and resulted = 57:43. HPLC conditions: Column Phenyl C6 150 × 3 mm. Injected volume: 5μL (1.5 mg/mL). Flow: 0.34 mL/min. Temp = 24 °C. Gradient elution: from H_2O/CH_3CN 90:10 to H_2O/CH_3CN 0:100 in 30 min. Detection: UV (220 μM). t_R: 23.9 min (major), 24.7 min (minor).

Major diastereomer: colourless oil. R_f = 0.17 (AcOEt/PE 1:2 + 1% MeOH). $[\alpha]_D$: - 36.7 (c 0.48, $CHCl_3$). ^{1}H NMR (300 MHz, d6-DMSO, 90 °C): δ 7.53 (2 H, dd, *J* = 8.0, 1.6 Hz.), 7.45-7.25 (8 H, m), 7.31 (1 H, d, *J* = 3.9 Hz., *H*-3 of thienyl), 6.99 (1 H, d, *J* = 3.9 Hz., *H*-4 of thienyl), 6.41 (broad s, N*H*), 5.19 (1 H, s, C*H*-C=O), 5.08 (1 H, d, *J* = 11.1 Hz., C*H*-Ph), 4.12 (1 H, t, *J* = 4.8 Hz., O*H*), 3.29 (1 H, ddd, *J* = 10.5, 4.2, 3.0 Hz., C*H*H-OH), 3.02 (1 H, dt, J_d= 10.5, J_t = 5.4 Hz., CH*H*-OH), 2.83 (2 H, q, *J* = 6.3 Hz., C*H*$_2$NH), 2.75-2.60 (1 H, m, C*H*(Me)-CH$_2$OH), 1.37-1.03 (6 H, m, C*H*$_2$C*H*$_2$C*H*$_2$CH$_3$), 0.92 (3 H, d, *J* = 6.6 Hz., C*H*$_3$CH), 0.86 (3 H, t, *J* = 7.0 Hz., C*H*$_3$CH$_2$). ^{13}C NMR (75 MHz, d6-DMSO, 90 °C): δ 167.0, 163.5 (*C*=O), 138.1, 137.9, 136.3, 131.1 (aromatic quat.), 128.1 (*C*-3 of thienyl), 129.2 (x 2), 128.8 (x 2), 127.7 (x 2), 127.3 (x 2), 127.2, 127.0 (*C*H of phenyls), 125.8 (*C*-4 of thienyl), 64.7 (*C*H-Ph), 64.1 (*C*H-C=O), 62.6 (*C*H$_2$OH), 38.3 (*C*H$_2$NH), 35.5 (*C*HCH$_3$), 28.9, 27.7, 21.0 (*C*H$_2$*C*H$_2$*C*H$_2$CH$_3$), 14.3 (*C*H$_3$CH), 13.0 (*C*H$_3$CH$_2$). I.r. (ATR): ν_{max} = 3414, 2961, 2930, 2872, 1659, 1602, 1529, 1496, 1429, 1325, 1259, 1078, 1031, 1010, 798, 751, 727, 698, 665, 638, 621 cm^{-1}. HRMS (ESI+). Calcd for $C_{28}H_{34}ClN_2O_3S$ (M + H$^+$) 513.1979; Found 513.1977.

Minor diastereomer: colourless oil. 0.22 (AcOEt/PE 1:2 + 1% MeOH). $[\alpha]_D$: - 146.81 (c 0.515, $CHCl_3$). Important note: the relative configuration of major and minor diastereomers was not determined.

5-Chloro-*N*-((*R* or *S*)-(cyclohexylamino)-3-methyl-2-oxobutan-2-yl)-*N*-((1*S*,2*S*)-3-hydroxy-2-isopropyl-1-phenylpropyl)thiophene-2-carboxamide (6h).

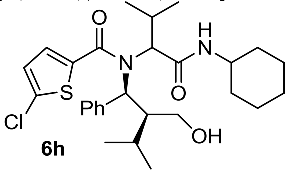

6h

It was prepared following the same procedures (A or B) used for **6a**. Overall yield (method A): 40%. Overall yield (method B): 70%. The diastereomeric ratio was determined by HPLC on the crude products and resulted = 78:22 (method A) or 77:23 (method B). HPLC conditions: Column Zorbax Eclipse XDB-C8 150 × 4.6 mm. Injected volume: 5 µL (1 mg/mL). Flow: 0.75 mL/min. Temp = 25 °C. Isocratic elution: H_2O/CH_3CN 35:65. Detection: UV (220 µM). t_R: 18.7 min (major), 21.6 min (minor).
Major diastereomer: colourless oil. R_f = 0.46 (AcOEt/PE 1:2 + 1% MeOH). $[\alpha]_D$:+86.4 (c 0.415, $CHCl_3$). ^{1}H NMR (300 MHz, d6-DMSO, 85 °C): δ 7.65 (1 H, d, *J* = 4.2 Hz., *H*-3 of thienyl), 7.52-7.44 (3 H, m, N*H* and phenyl C*H*), 7.32-7.18 (3 H, m, phenyl C*H*), 7.10 (1 H, d, *J* = 4.2 Hz., *H*-4 of thienyl), 5.19 (1 H, s, C*H*-Ph), 3.90-3.40 (1 H, very broad s, O*H*), 3.61 (1 H, d, *J* = 9.9 Hz., C*H*-C=O), 3.40-3.25 (1 H, m, C*H*NH), 3.34 (1 H, dd, *J* = 11.0, 3.0 Hz., C*H*H-OH), 3.16 (1 H, dd, *J* = 6.3 Hz., C*H*H-OH), 2.65-2.53 (1 H, m, C*H*(*i*Pr)-CH_2OH), 2.40-2.20 (1 H, m, $(CH_3)_2$C*H*-CHC=O), 2.07 (1 H, d of heptuplets, J_d = 2.0, J_h = 6.6 Hz., $(CH_3)_2$C*H*-$CHCH_2$OH), 1.65-0.85 (10 H, m, CH_2 of cyclohexyl), 1.07 (3 H, d, *J* = 6.9 Hz., $(CH_3)_2$CH), 0.98 (3 H, d, *J* = 6.9 Hz., $(CH_3)_2$CH), 0.86 (3 H, d, *J* = 6.6 Hz., $(CH_3)_2$CH) 0.72 (3 H, d, *J* = 6.6 Hz., $(CH_3)_2$CH). ^{13}C NMR (75 MHz, d6-DMSO, 85 °C): δ 168.3, 164.9 (*C*=O), 138.8, 138.5, 131.3 (aromatic quat.), 128.9 (*C*-3 of thienyl), 128.9 (x 2), 127.1 (x 2), 126.7 (*C*H of phenyl), 126.0 (*C*-4 of thienyl), 69.4 (*C*H-C=O), 65.4 (*C*H-Ph), 58.3 (*C*H_2OH), 47.0 (*C*HNH), 44.8 (*C*HCH_2OH), 31.2 (x 2), 24.7, 23.6 (x 2) (*C*H_2 of cyclohexyl), 27.8 $(CH_3)_2$*C*H-CHC=O), 26.1 $(CH_3)_2$*C*H-$CHCH_2$OH), 22.4, 19.6, 19.4, 16.8 (*C*H_3). I.r. (ATR): ν_{max}= 3397, 3276, 2964, 2930, 2855, 1722, 1646, 1542, 1436, 1362, 1330, 1260, 1090, 1063, 1031, 1005, 800, 735, 702, 639, 625, 610 cm^{-1}. HRMS (ESI+). Calcd for $C_{28}H_{40}ClN_2O_3S$ (M + H^+) 519.2448; Found 519.2452.
Minor diastereomer: colourless oil. R_f = 0.53 (AcOEt/PE 1:2 + 1% MeOH). Important note: the relative configuration of major and minor diastereomers was not determined.

5-Chloro-*N*-((1*S*,2*S*)-1-(2-benzyloxyphenyl)-3-hydroxy-2-isopropylpropyl)-*N*-((*R* or *S*)-(cyclohexylamino)-3-methyl-2-oxobutan-2-yl)thiophene-2-carboxamide (6i).

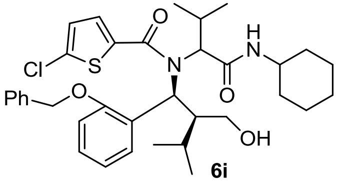

6i

It was prepared following the same procedures (A or B) used for **6a**. Overall yield (method A): 30%. Overall yield (method B): 54%. The diastereomeric ratio was determined by HPLC on the crude products and resulted = 64:36 (method A) or 69:31 (method B). HPLC conditions: Column Zorbax Eclipse XDB-C8 150 × 4.6 mm. Injected volume: 5 µL (1 mg/mL). Flow: 0.75 mL/min. Temp = 25 °C. Gradient elution: from H_2O/CH_3CN 90:10 to H_2O/CH_3CN 0:100 in 20 min. Detection: UV (220 µM). t_R: 22.0 min (major), 22.7 min (minor).

Major diastereomer: colourless oil. R_f = 0.56 (AcOEt/PE 1:1 + 1% MeOH). $[\alpha]_D$:+124.0 (c 0.375, $CHCl_3$). ^{1}H NMR (300 MHz, d6-DMSO, 85 °C): δ 7.63 (1 H, dd, *J* = 8.4, 2.1 Hz., *H*-6 of aryl), 7.46 (1 H, d, *J* = 3.9 Hz., *H*-3 of thienyl), 7.42-7.31 (5 H, m, C*H* of benzyl), 7.21 (1 H, dt, J_t = 7.8, J_d = 1.5 Hz., *H*-4 of aryl), 7.17-7.10 (1 H, broad d, N*H*), 7.00 (1 H, d, *J* = 7.8 Hz., *H*-3 of aryl), 6.89 (1 H, t, *J* = 7.5 Hz., *H*-5 of aryl), 6.88 (1 H, d, *J* = 3.9 Hz., *H*-4 of thienyl),
5.52 (1 H, d, *J* = 10.8 Hz., C*H*-Ar), 5.07 and 4.99 (2 H, AB syst., *J* = 12.2 Hz., CH_2Ph), 3.78 (1 H, broad t, O*H*), 3.56 (1 H, d, *J* = 9.3 Hz., C*H*-C=O), 3.35-3.18 (1 H, m, C*H*NH), 3.31 (1 H, t, *J* = 14.8, CH_2OH), 2.56-2.35 (2 H, m, C*H*(*i*Pr)-CH_2OH and $(CH_3)_2$C*H*-CHC=O), 2.02 (1 H, d of heptuplets, J_d = 2.0, J_h = 6.6 Hz., $(CH_3)_2$C*H*-CHCH_2OH), 1.75-0.85 (10 H, m, CH_2 of cyclohexyl), 1.03 (3 H, d, *J* = 6.9 Hz., (CH_3)$_2$CH), 0.89 (3 H, d, *J* = 6.6 Hz., (CH_3)$_2$CH), 0.88 (3 H, d, *J* = 6.6 Hz., (CH_3)$_2$CH) 0.82 (3 H, d, *J* = 6.9 Hz., (CH_3)$_2$CH). ^{13}C NMR (75 MHz, d6-DMSO, 85 °C): δ 168.8, 165.3 (*C*=O), 155.8 (*C*-2 of aryl), 138.6, 136.4, 131.3, 126.6 (aromatic quat.), 130.0 (*C*-6 of aryl), 128.5 (*C*-3 of thienyl), 128.2 (*C*-4 of aryl), 127.7 (x 2), 127.2, 127.0 (x 2) (aromatic *C*H of benzyl), 126.3 (*C*-4 of thienyl), 119.9 (*C*-5 of Ar), 111.9 (*C*-3 of aryl), 69.6 (*C*H_2Ph), 69.2 (*C*H-C=O), 58.3 (*C*H_2OH), 58.2 (*C*H-Ar), 46.27 (*C*HNH), 45.2 (*C*HCH_2OH), 31.2, 31.1 (x 2), 24.6, 23.4 (x 2) (*C*H_2 of cyclohexyl), 27.8 $(CH_3)_2$*C*H-CHC=O), 26.2 $(CH_3)_2$*C*H-CHCH_2OH), 22., 20.0, 19.8, 16.7 (*C*H_3). I.r. (ATR): ν_{max} = 3277, 2963, 2929, 2854, 1739, 1646, 1535, 1491, 1449, 1433, 1363, 1304, 1259, 1231, 1094, 1054, 1008, 799, 755, 737, 696, 630 cm^{-1}. HRMS (ESI+). Calcd for $C_{35}H_{45}ClN_2NaO_4S$ (M + Na^+) 647.2686; Found 647.2698.
Minor diastereomer: colourless oil. R_f = 0.70 (AcOEt/PE 1:1 + 1% MeOH). Important note: the relative configuration of major and minor diastereomers was not determined.

N-((1*S*,2*S*)-2-benzyl-1-(2-benzyloxyphenyl)-3-hydroxypropyl)-2-((benzyloxycarbonyl)amino))-*N*-((*R* or *S*)-(*tert*-butylamino)-3-methyl-2-oxobutan-2-yl)acetamide (6j).

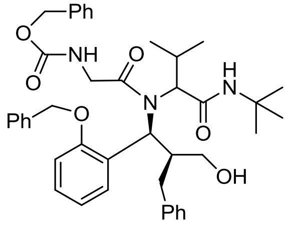

6j

It was prepared following the same procedures (A or B) used for **6a**. Overall yield (method A): 48%. Overall yield (method B): 77%. The diastereomeric ratio was determined by HPLC on the crude products and resulted = 80:20 (method A) or 81:19 (method B). HPLC conditions: Column Phenyl C6 150 × 3 mm. Injected volume: 5 µL (0.5 mg/mL). Flow: 0.34 mL/min. Temp = 31 °C. Gradient elution: from H_2O/CH_3CN 90:10 to H_2O/CH_3CN 0:100 in 20 min. Detection: UV (220 µM). t_R: 20.1 min (major), 21.1 min (minor).
Major diastereomer: colourless oil. R_f = 0.35 (AcOEt/PE 1:2 + 1% MeOH). $[\alpha]_D$: + 45.8 (c 0.5, $CHCl_3$). ^{1}H NMR (300 MHz, d6-DMSO, 110 °C): δ 7.51 (1 H, d, *J* = 7.5 Hz., *H*-6 of aryl), 7.39 (1 H, t, *J* = 7.5 Hz., *H*-4 of aryl), 7.37-7.15 (10 H, m, C*H* of benzyl), 7.02 (1 H, d, *J* = 8.1 Hz., *H*-3 of aryl), 6.89 (1 H, t, *J* = 7.5 Hz., *H*-5 of aryl), 6.69 (1 H, broad s, N*H* amide), 6.50 (1 H, broad s, N*H* urethane), 5.40 (1 H, d, *J* = 10.2 Hz., C*H*-Ar), 5.19 (2 H, s, CH_2Ph), 5.05 (2 H, s, CH_2Ph), 4.22 and 4.18 (2 H, AB part of ABX syst., J_{AB} = 15.9 J_{AX} = 5.2, J_{BX} = 4.4 Hz., CH_2NHCbz), 3.93 (1 H, broad s, O*H*), 3.49 (1 H, d, *J* = 8.1 Hz., C*H*-C=O), 3.28-3.08 (2 H, m, CH_2OH), 3.00-2.85 (2 H, m, C-CH_2Ph), 2.82-2.68 (1 H, m, C*H*(Bn)-CH_2OH), 2.60-2.40 (1 H, m, $(CH_3)_2$C*H*), 0.98 (9 H, s, (CH_3)$_3$C), 0.96 (3 H, d, *J* = 6.6 Hz., CH_3), 0.94 (3 H, d, *J* = 6.6 Hz., CH_3). ^{13}C NMR (75 MHz, d6-DMSO, 85 °C): δ 170.9, 168.5 (broad), 156.3 (*C*=O), 155.7 (*C*-2 of aryl), 140.0 (broad), 136.6, 136.4, 125.1 (aromatic quat.), 130.2, 128.4 (x 2), 128.1, 127.9 (x 2), 127.6 (x 2), 127.5 (x 2), 127.3 (x 2), 127.2, 127.0, 126.9 (x 2), 125.1 (*C*H of benzyls, and *C*-4 and *C*-6 of aryl), 119.8 (*C*-5 of Ar), 111.9 (*C*-3 of aryl), 69.5, 69.1, 65.0 (O*C*H_2Ph, *C*H-C=O), 57.5, 55.7 (broad) (*C*H_2OH, *C*H-Ar, *C*N), 48.9, 43.3 (*C*H_2NH, *C*HCH_2OH), 32.6 (C-*C*H_2Ph), 28.8 $(CH_3)_2$*C*H), 27.6 (C(*C*H_3)$_3$), 21.5 (broad), 19.6 (*C*H_3). I.r. (ATR): ν_{max} = 3311, 3032, 2965, 1721, 1662,

1601, 1544, 1497, 1453, 1223, 1162, 1050, 1025, 1007, 735, 697, 670, 619, 609. cm^{-1}. HRMS (ESI+). Calcd for $C_{42}H_{52}N_3NO_6$ (M + H^+) 6494.3856; Found 694.3863.

Minor diastereomer: colourless oil. R_f = 0.42 (AcOEt/PE 1:2 + 1% MeOH). Important note: the relative configuration of major and minor diastereomers was not determined.

References

1. Yang, J. W.; Pan, S. C.; List, B. *Org. Synth.* **2009**, *86*, 11-17.
2. Morana, F.; Basso, A.; Riva, R.; Rocca, V.; Banfi, L. *Chem. Eur. J.* **2013**, *19*, 4563-4569.
3. Joannesse, C.; Johnston, C. P.; Morrill, L. C.; Woods, P. A.; Kieffer, M.; Nigst, T. A.; Mayr, H.; Lebl, T.; Philp, D.; Bragg, R. A.; Smith, A. D. *Chem. Eur. J.* **2012**, *18*, 2398-2408.
4. Yang, J. W.; Stadler, M.; List, B. *Angew. Chem, Int. Ed.* **2007**, *46*, 609-611.
5. Joannesse, C.; Johnston, C. P.; Concellón, C.; Simal, C.; Philp, D.; Smith, A. D. *Angew. Chem, Int. Ed.* **2009**, *48*, 8914-8918.
6. Banfi, L.; Basso, A.; Giardini, L.; Riva, R.; Rocca, V.; Guanti, G. *Eur. J. Org. Chem.* **2011**, 100-109.

2) ^{1}H and ^{13}C NMR spectra of all new compounds

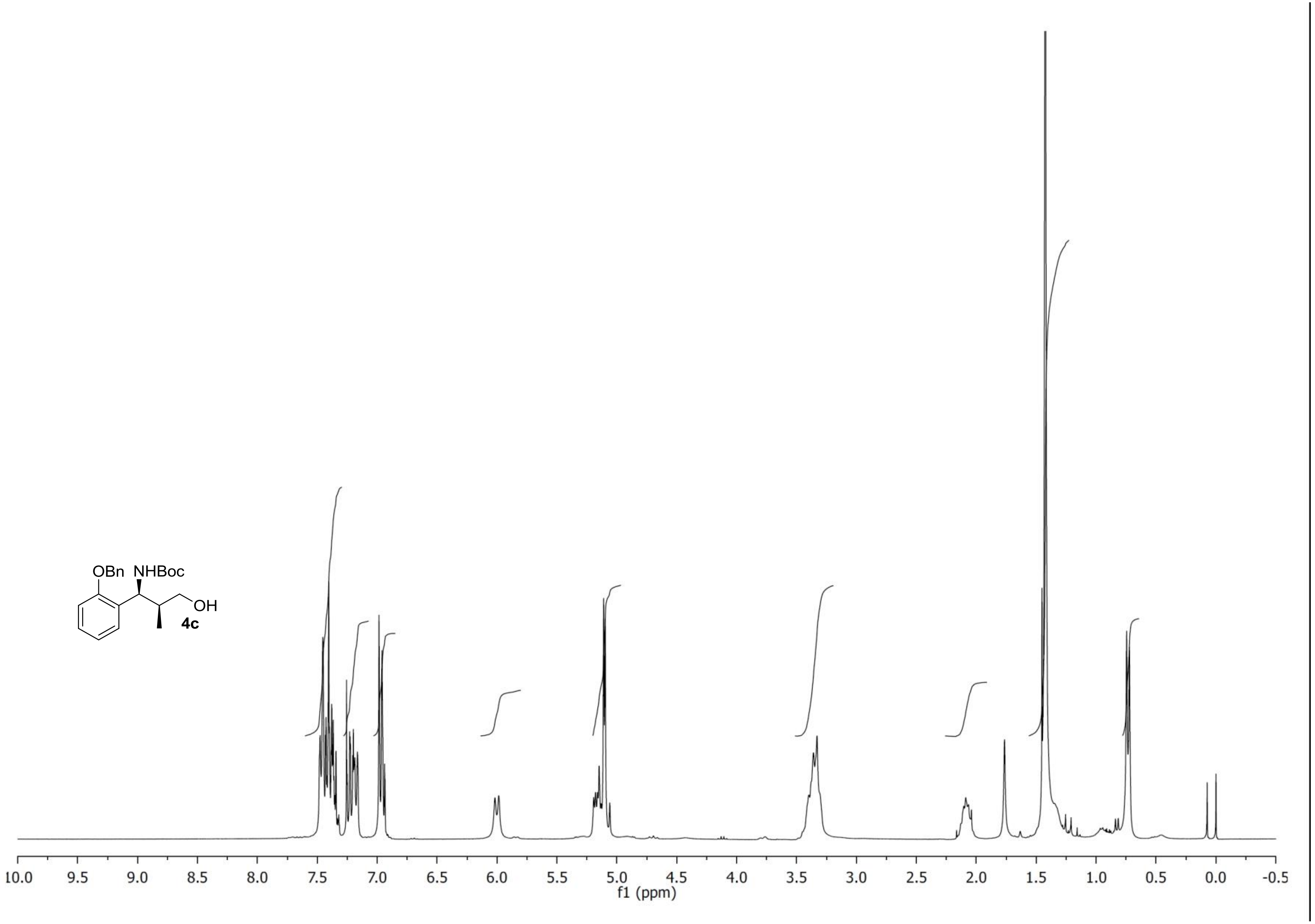

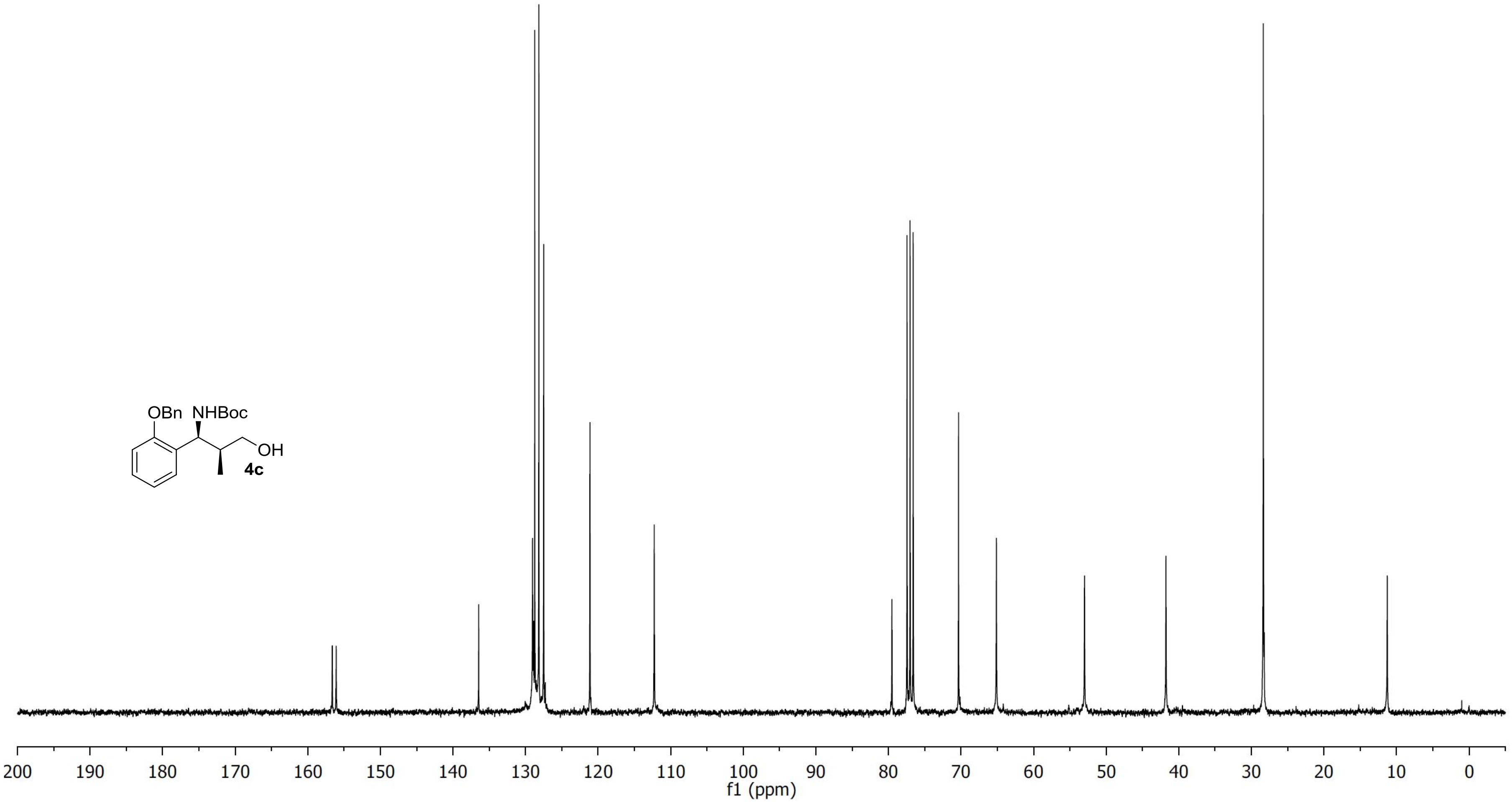
OBn
NHBoc
OH
4c
200 190 180 170 160 150 140 130 120 110 100 90 80 70 60 50 40 30 20 10 0
f1 (ppm)

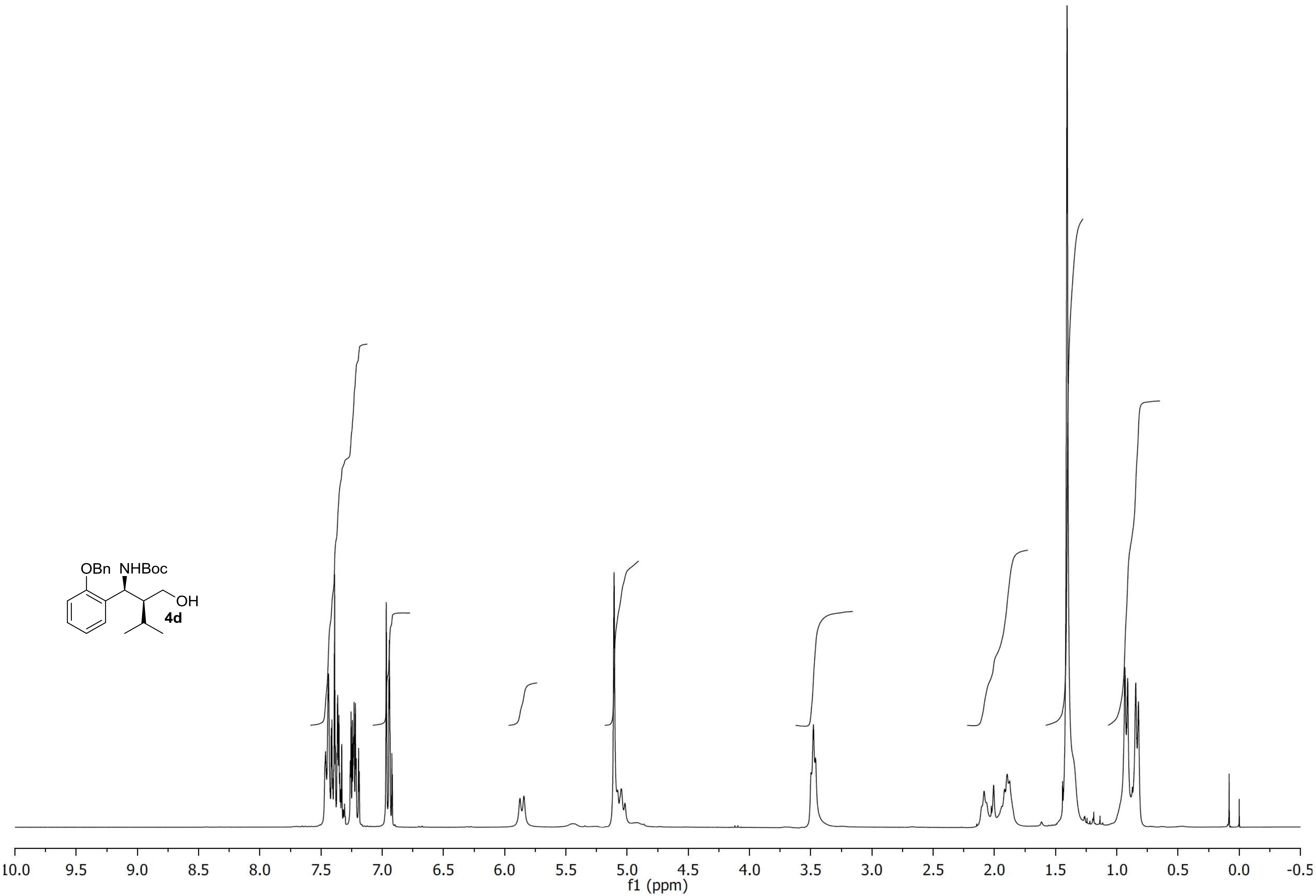
OBn
NHBoc
OH
4d
10.0
9.5
9.0
8.5
8.0
7.5
7.0
6.5
6.0
5.5
5.0
4.5
4.0
3.5
3.0
2.5
2.0
1.5
1.0
0.5
0.0
-0.5
f1 (ppm)

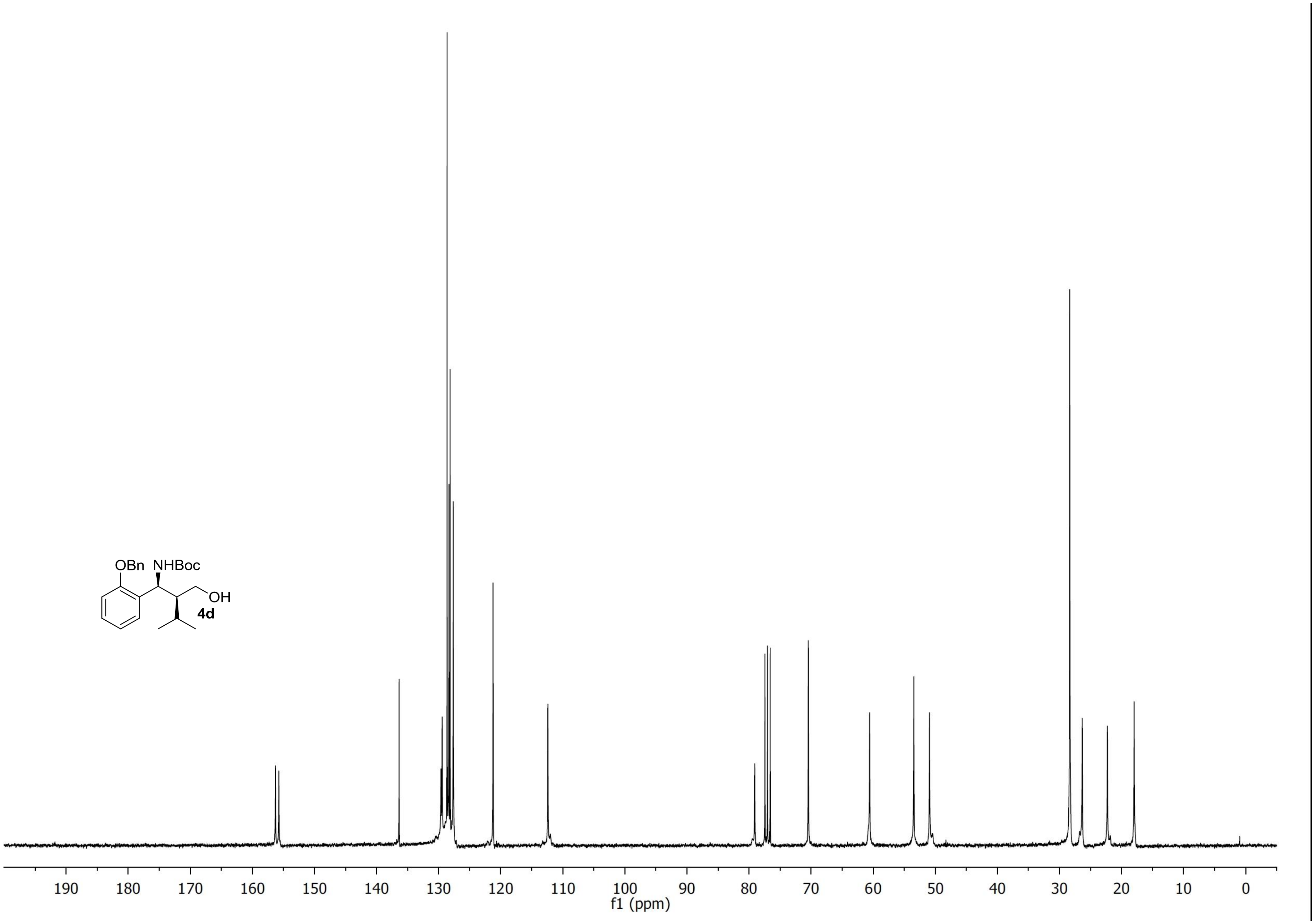
OBn
NHBoc
OH
4d
190 180 170 160 150 140 130 120 110 100 90 80 70 60 50 40 30 20 10 0
f1 (ppm)

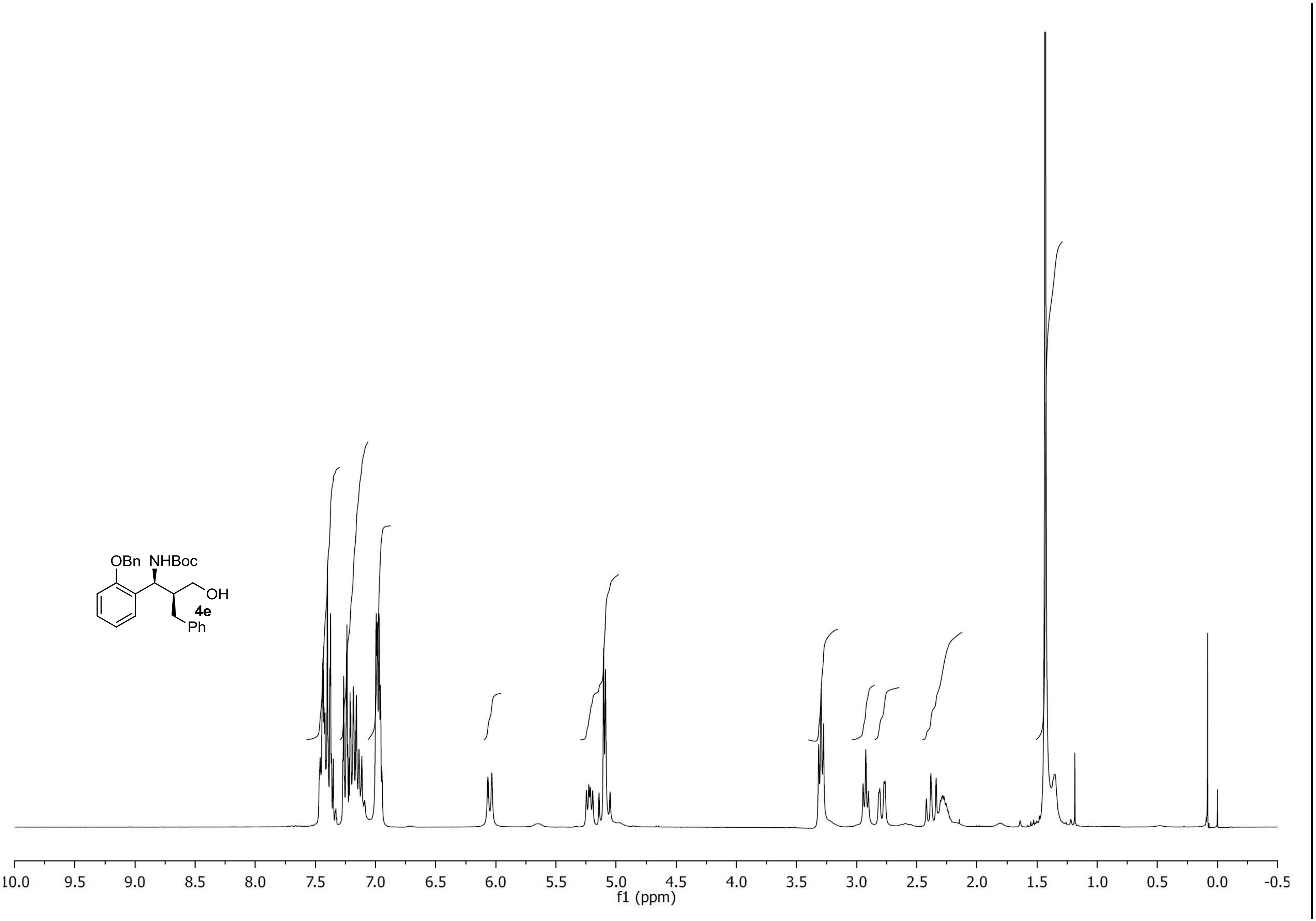
OBn
NHBoc
OH
4e
Ph
10.0 9.5 9.0 8.5 8.0 7.5 7.0 6.5 6.0 5.5 5.0 4.5 4.0 3.5 3.0 2.5 2.0 1.5 1.0 0.5 0.0 -0.5
f1 (ppm)

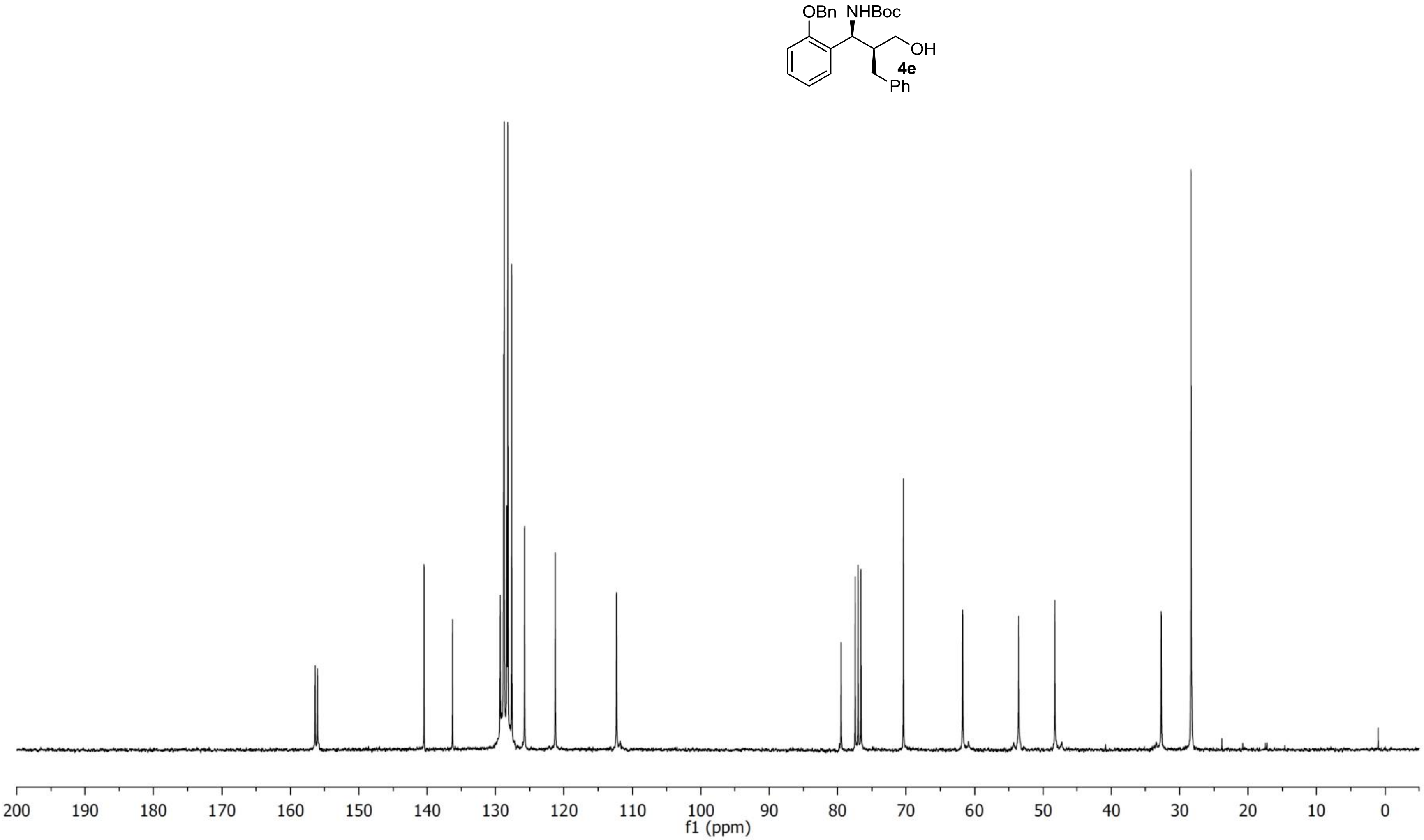
OBn
NHBoc
OH
4e
Ph
200 190 180 170 160 150 140 130 120 110 100 90 80 70 60 50 40 30 20 10 0
f1 (ppm)

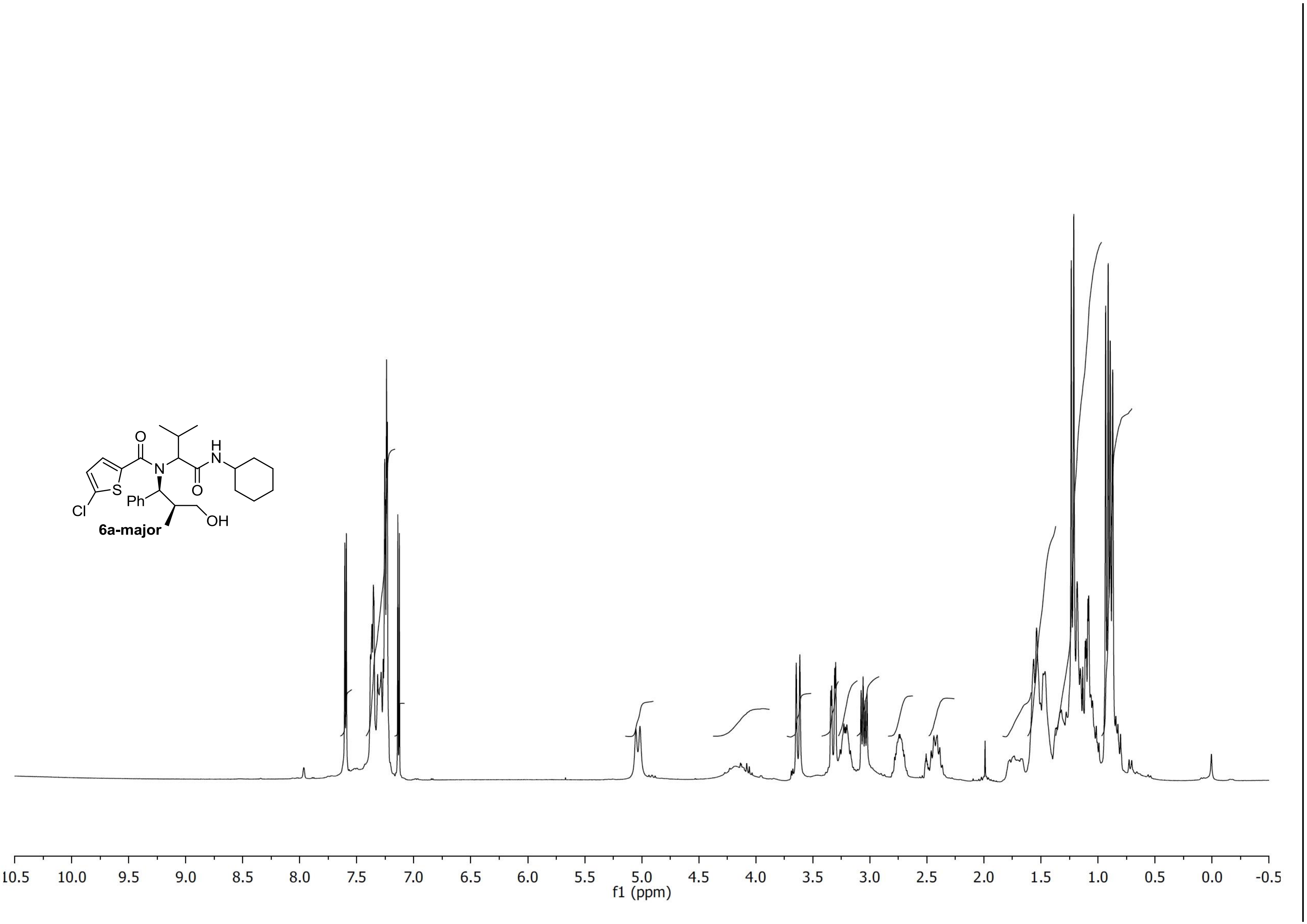

O
N
H
S
Ph
Cl
OH
6a-major
10.5
10.0
9.5
9.0
8.5
8.0
7.5
7.0
6.5
6.0
5.5
5.0
4.5
4.0
3.5
3.0
2.5
2.0
1.5
1.0
0.5
0.0
-0.5
f1 (ppm)

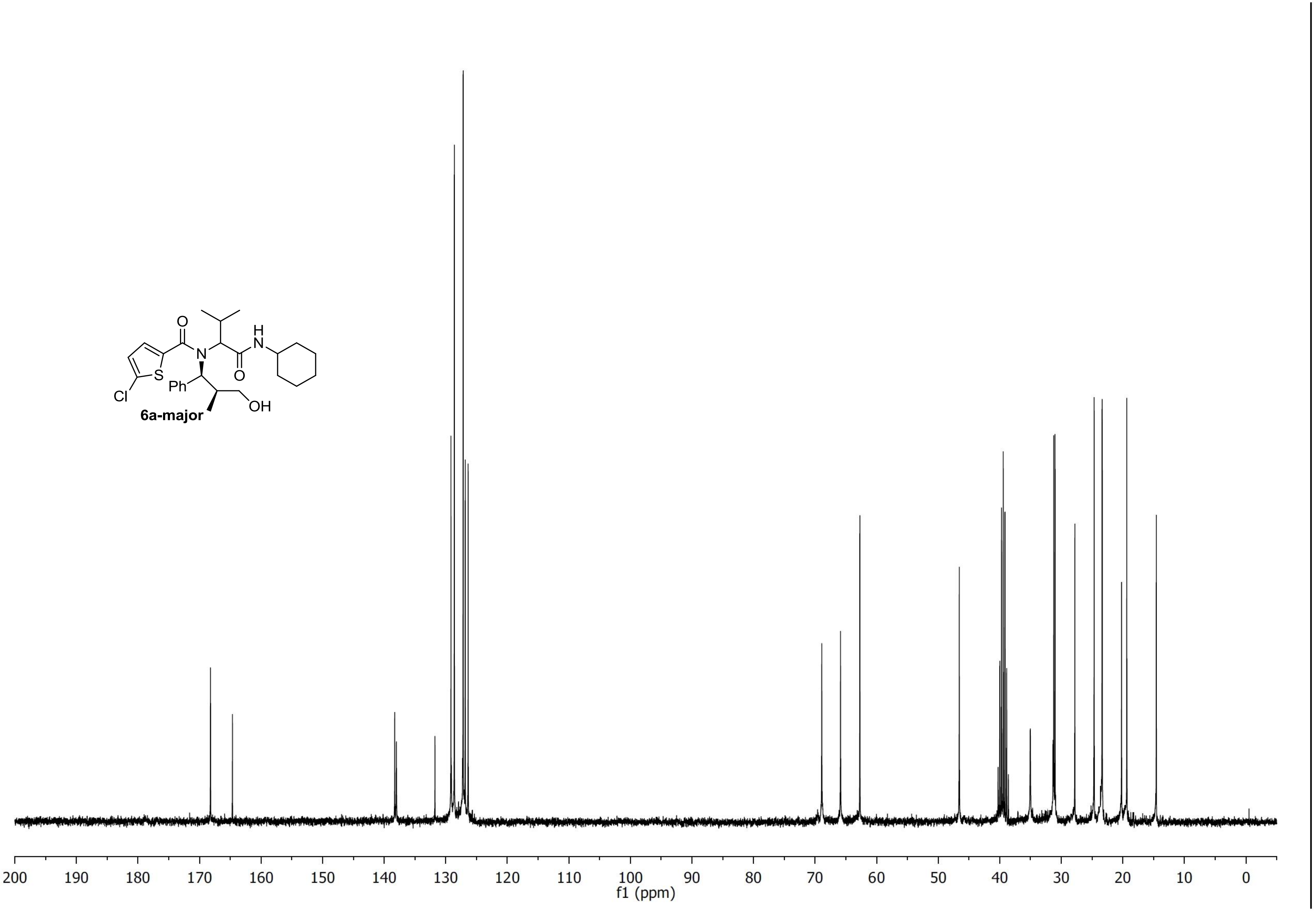
O
H
N
N
O
S
Ph
Cl
OH
200 190 180 170 160 150 140 130 120 110 100 90 80 70 60 50 40 30 20 10 0
f1 (ppm)

6a-major

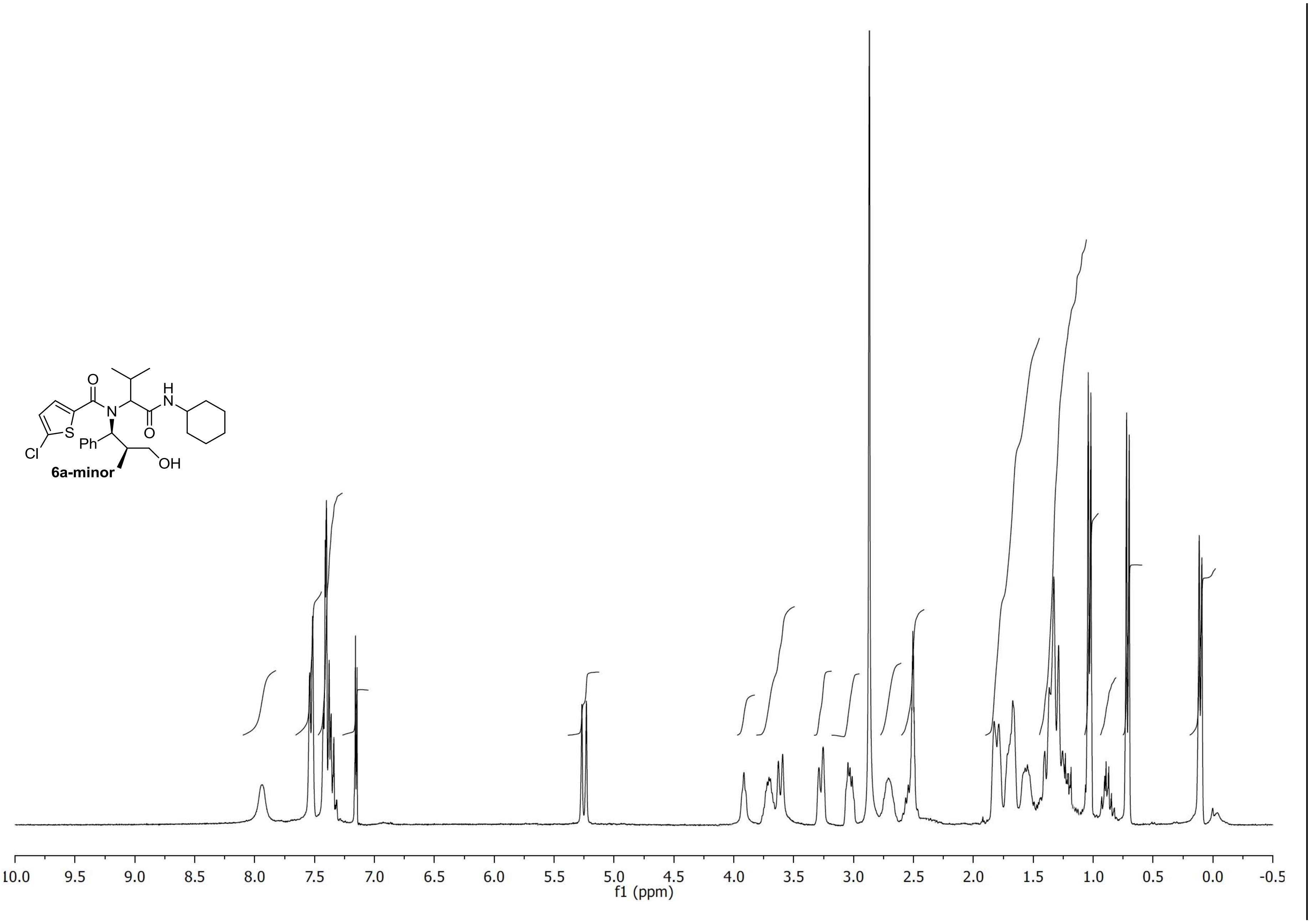
6a-minor
10.0 9.5 9.0 8.5 8.0 7.5 7.0 6.5 6.0 5.5 5.0 4.5 4.0 3.5 3.0 2.5 2.0 1.5 1.0 0.5 0.0 -0.5
f1 (ppm)

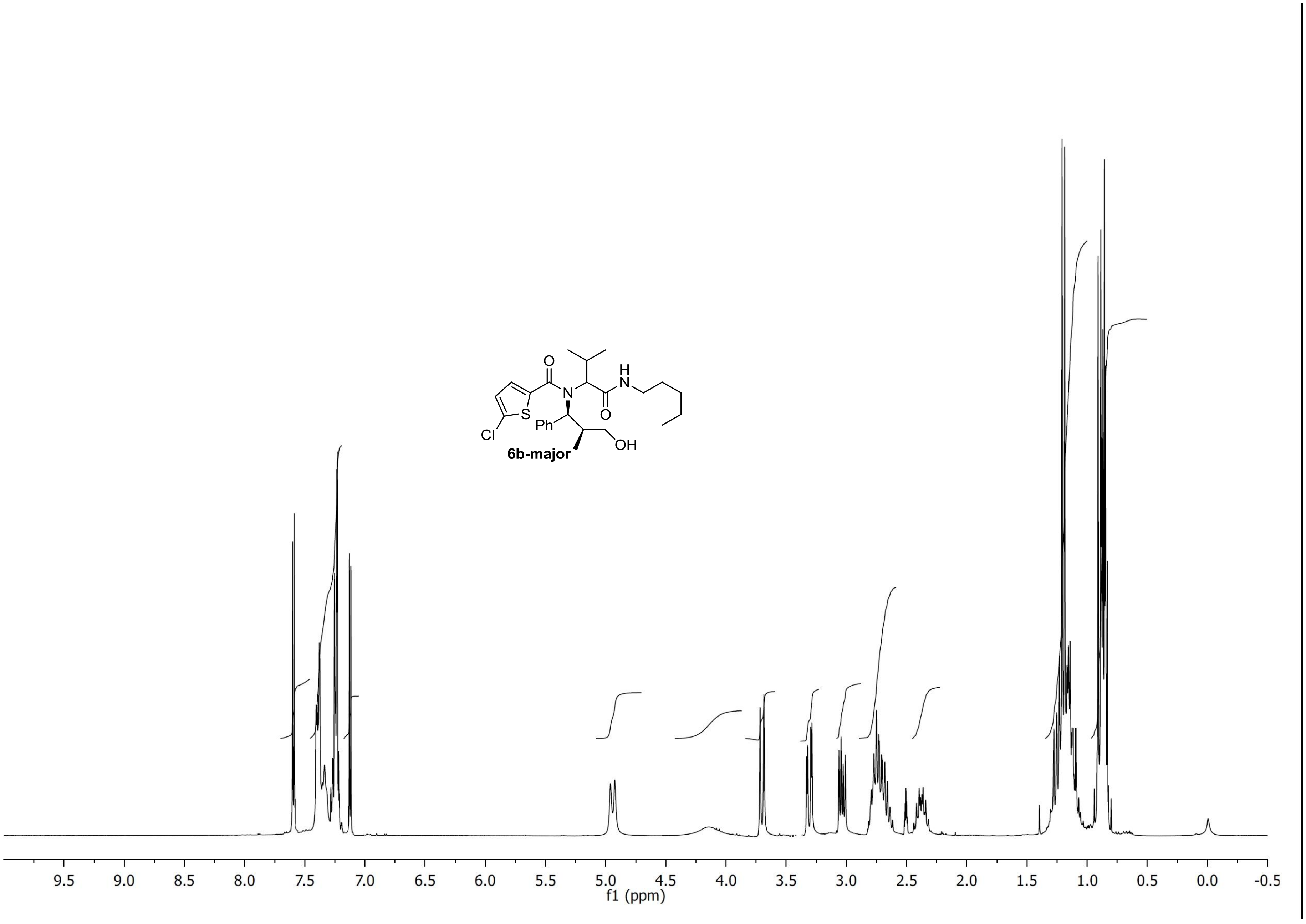
O
N
H
S
Cl
Ph
OH
6b-major
9.5 9.0 8.5 8.0 7.5 7.0 6.5 6.0 5.5 5.0 4.5 4.0 3.5 3.0 2.5 2.0 1.5 1.0 0.5 0.0 -0.5
f1 (ppm)

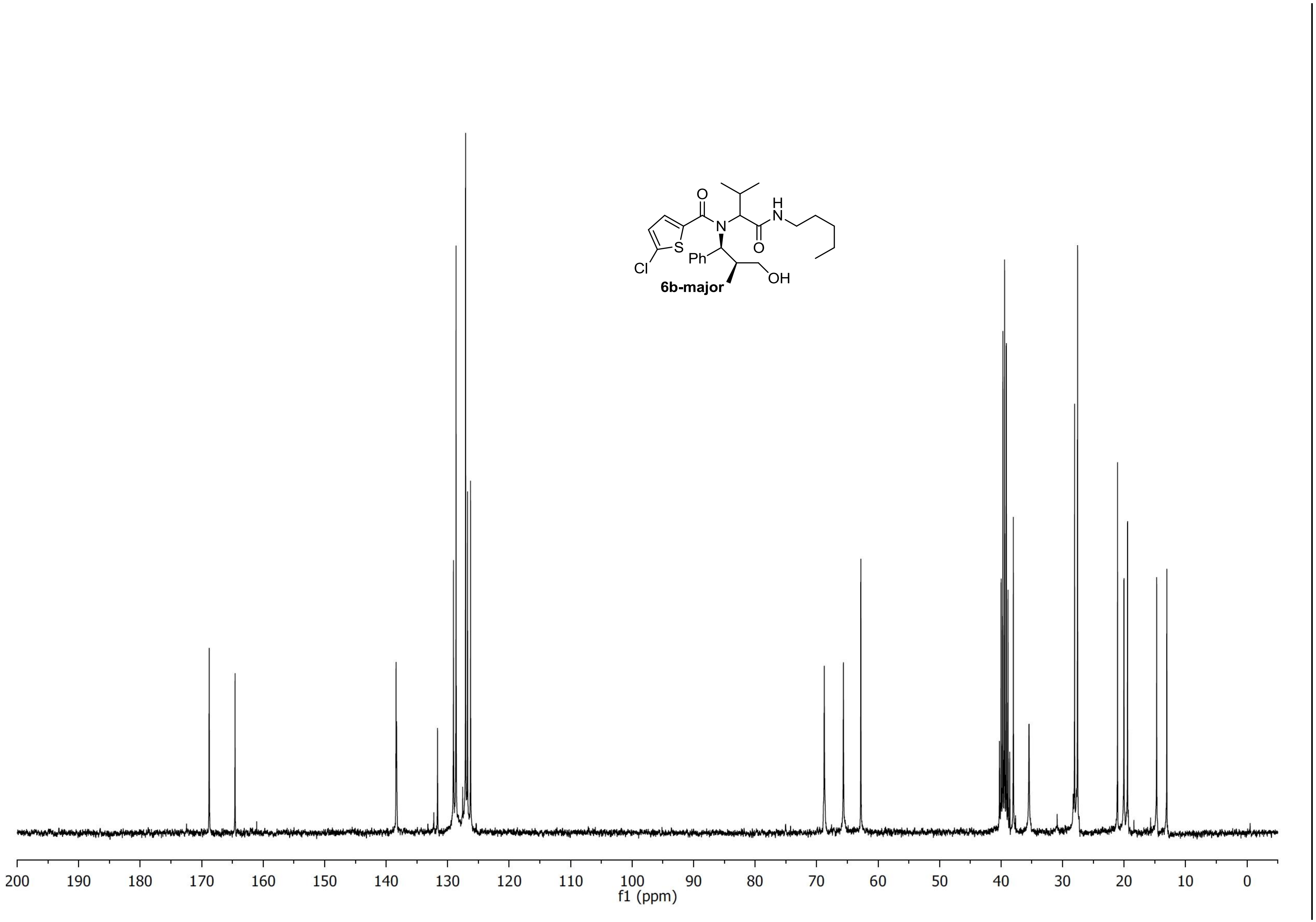

O
H
N
N
O
S
Ph
Cl
OH
6b-major
200
190
180
170
160
150
140
130
120
110
100
90
80
70
60
50
40
30
20
10
0
f1 (ppm)

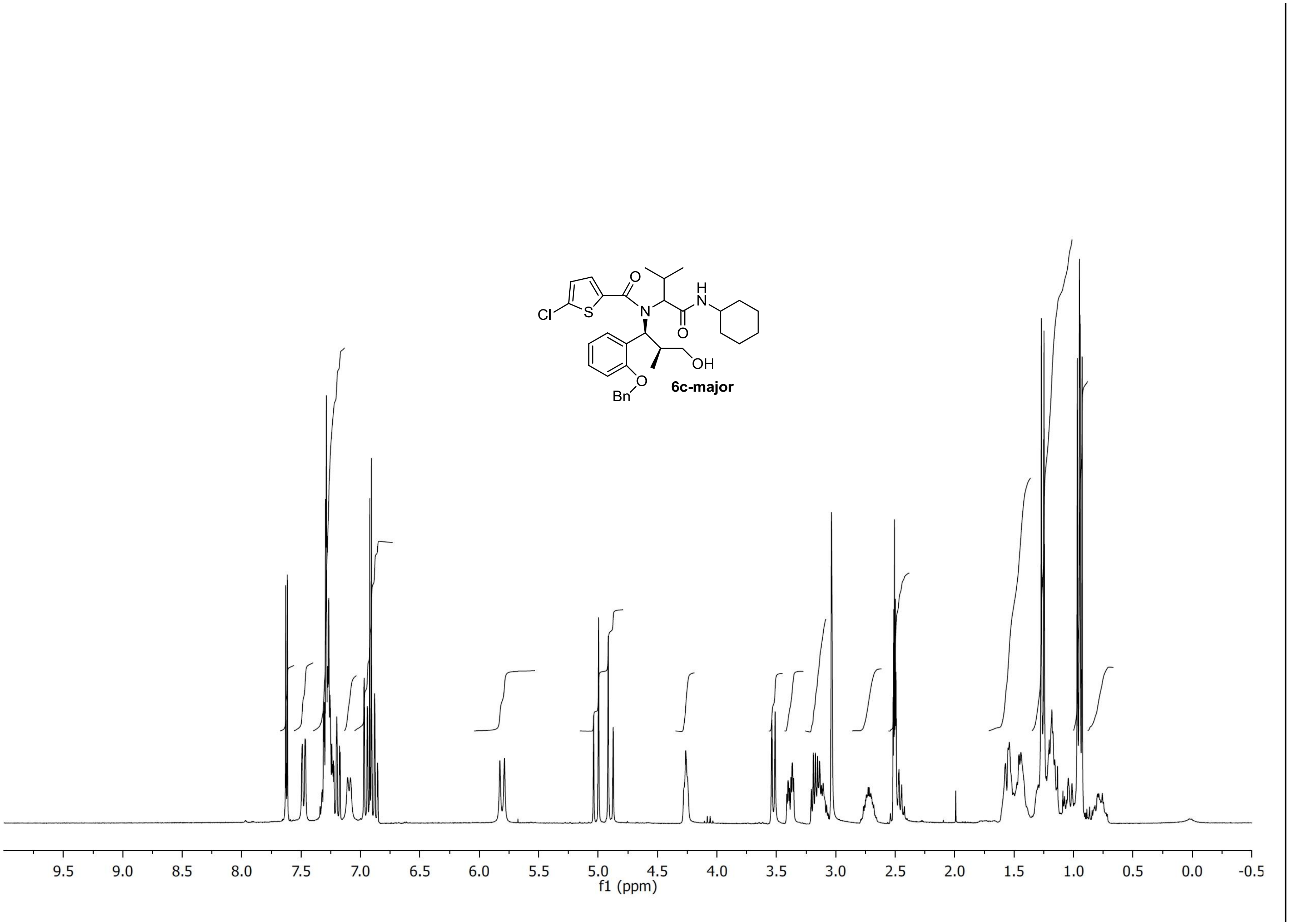

Cl
S
O
N
H
N
O
OH
O
Bn
6c-major
9.5 9.0 8.5 8.0 7.5 7.0 6.5 6.0 5.5 5.0 4.5 4.0 3.5 3.0 2.5 2.0 1.5 1.0 0.5 0.0 -0.5
f1 (ppm)

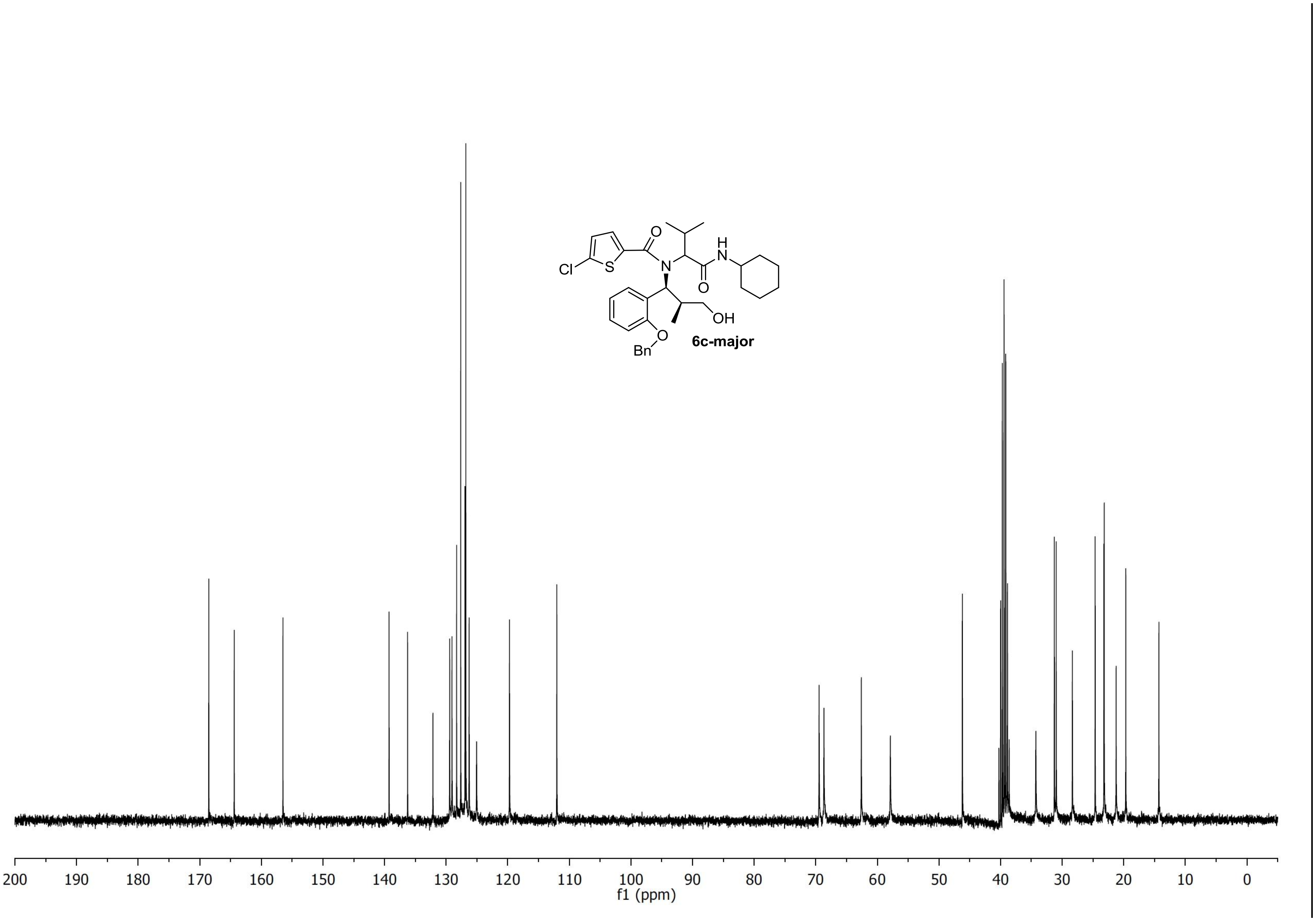
Cl
S
O
N
H
N
O
OH
O
Bn
6c-major
200 190 180 170 160 150 140 130 120 110 100 90 80 70 60 50 40 30 20 10 0
f1 (ppm)

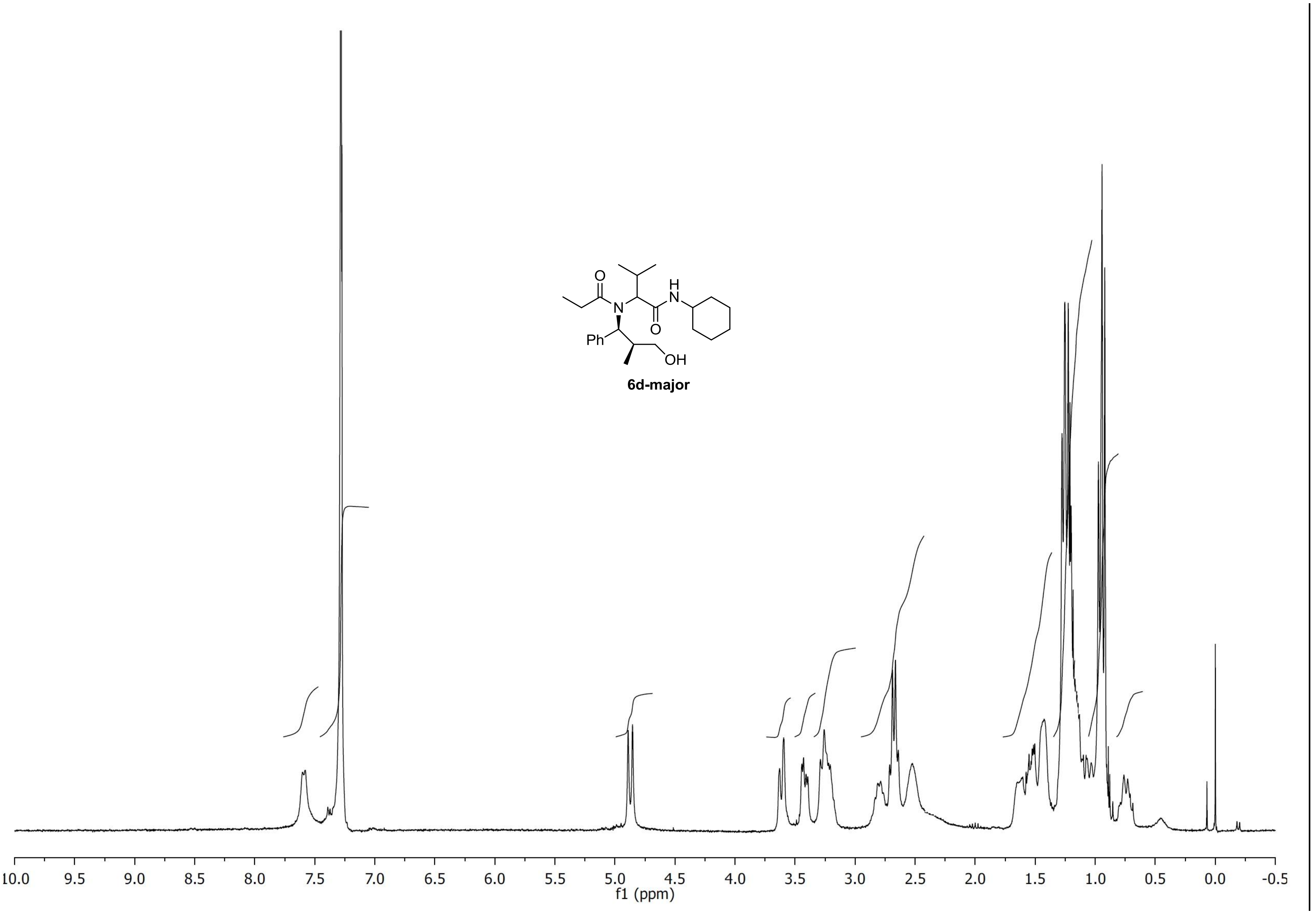

O
H
N
N
O
Ph
OH
6d-major
10.0
9.5
9.0
8.5
8.0
7.5
7.0
6.5
6.0
5.5
5.0
4.5
4.0
3.5
3.0
2.5
2.0
1.5
1.0
0.5
0.0
-0.5
f1 (ppm)

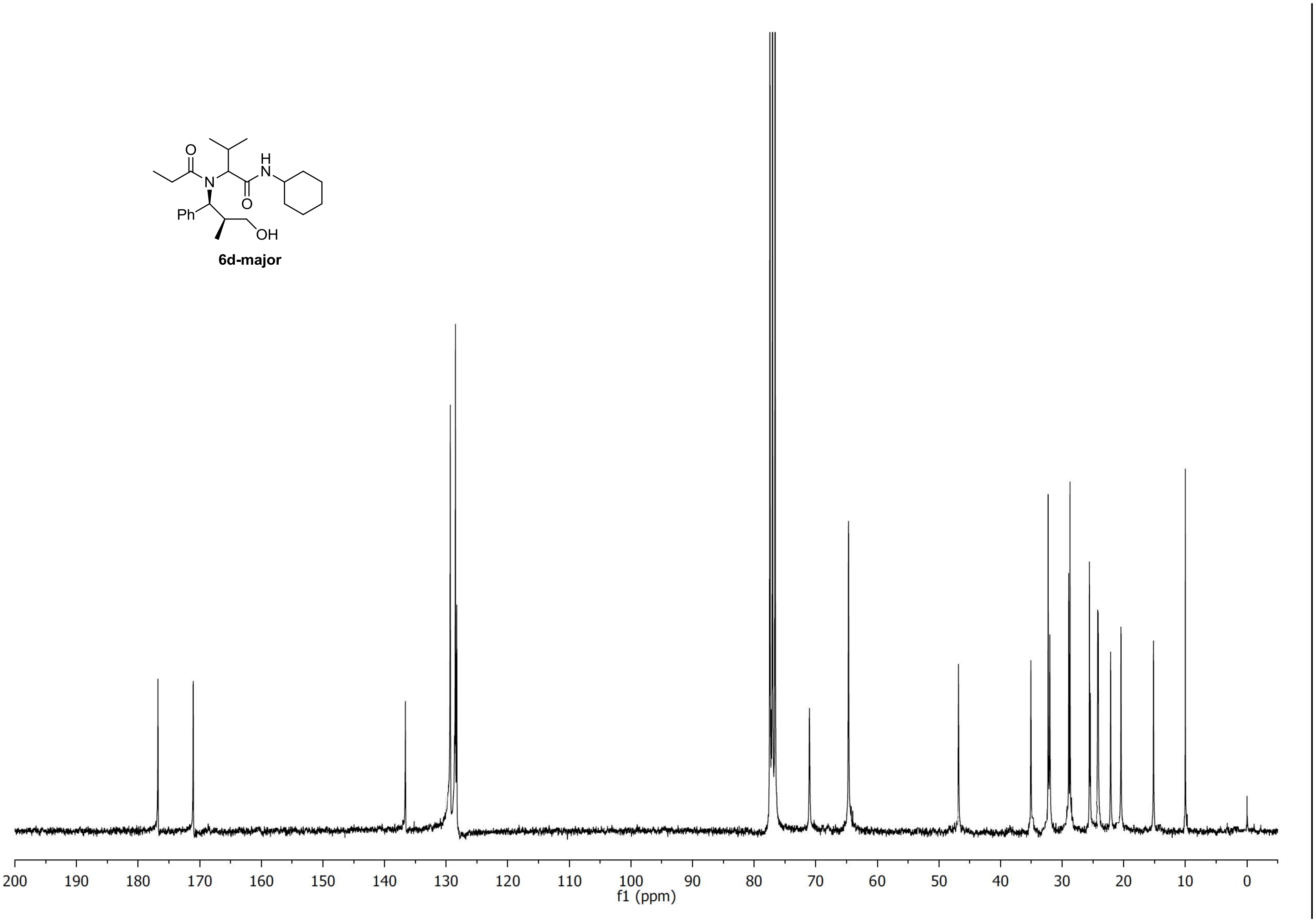
O
H
N
Ph
O
OH
6d-major
200 190 180 170 160 150 140 130 120 110 100 90 80 70 60 50 40 30 20 10 0
f1 (ppm)

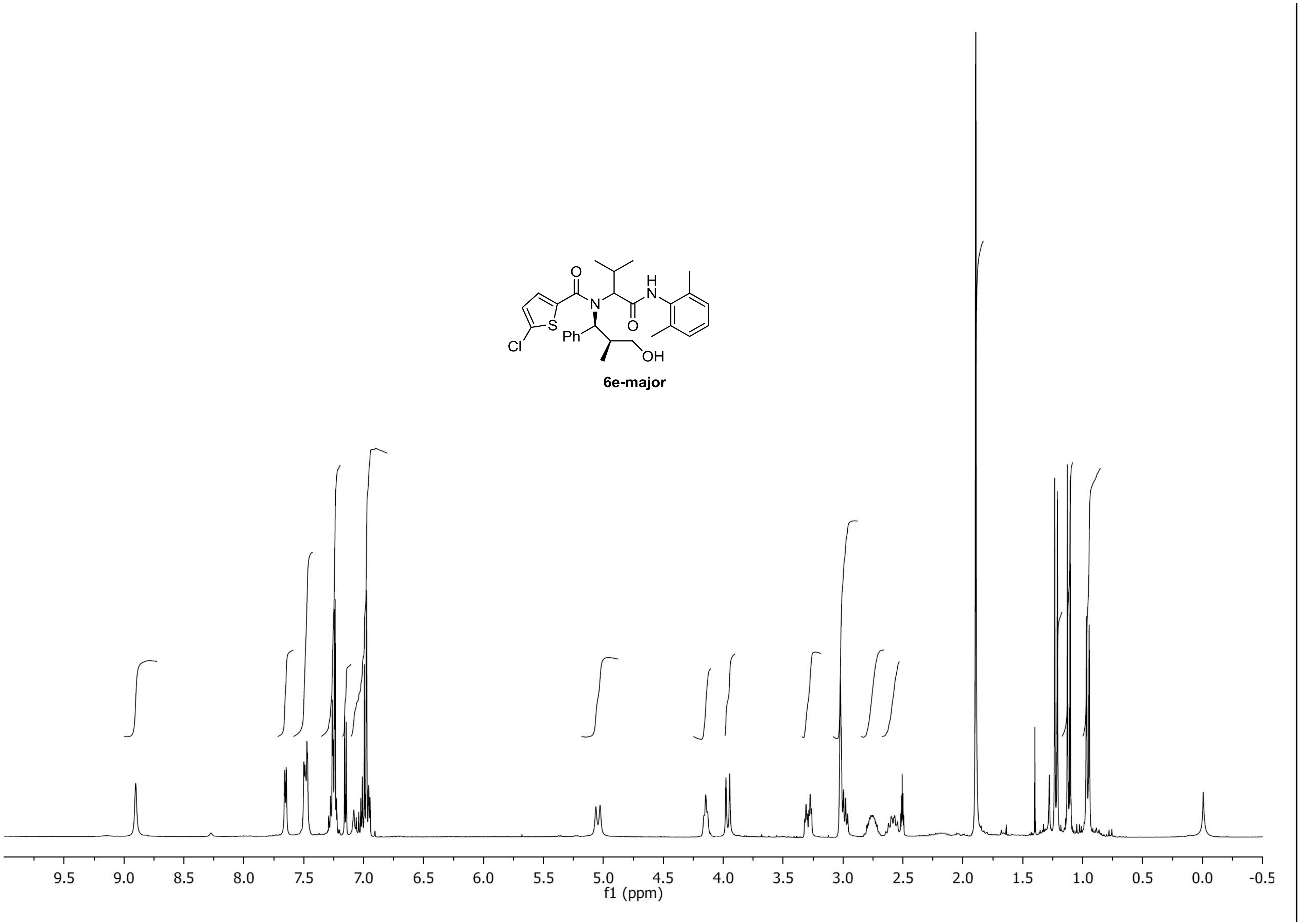
O
H
N
N
S
Cl
Ph
O
OH
6e-major
9.5
9.0
8.5
8.0
7.5
7.0
6.5
6.0
5.5
5.0
4.5
4.0
3.5
3.0
2.5
2.0
1.5
1.0
0.5
0.0
-0.5
f1 (ppm)

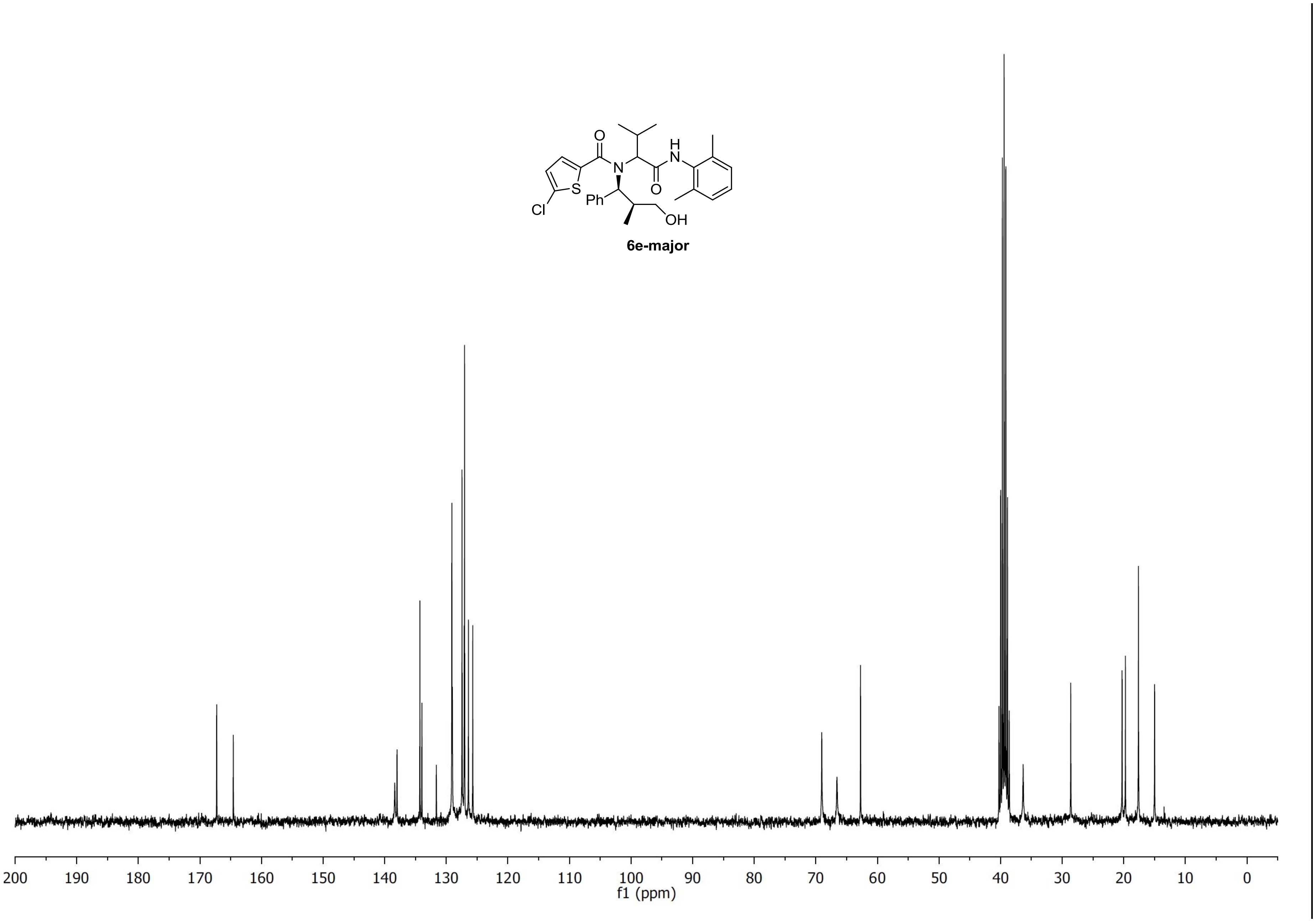
O
N
H
N
O
S
Cl
Ph
OH
6e-major
200 190 180 170 160 150 140 130 120 110 100 90 80 70 60 50 40 30 20 10 0
f1 (ppm)

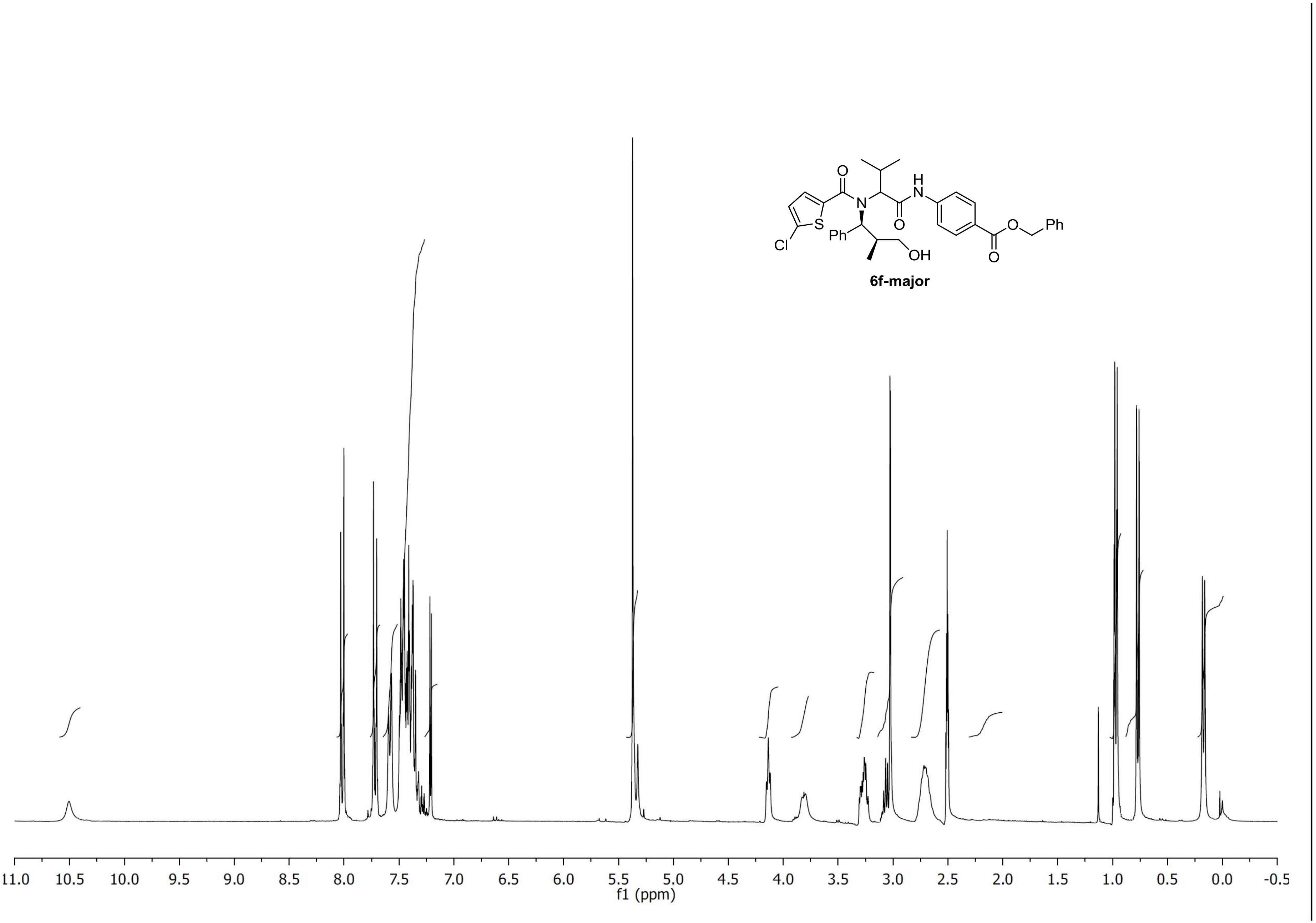
6f-major
f1 (ppm)

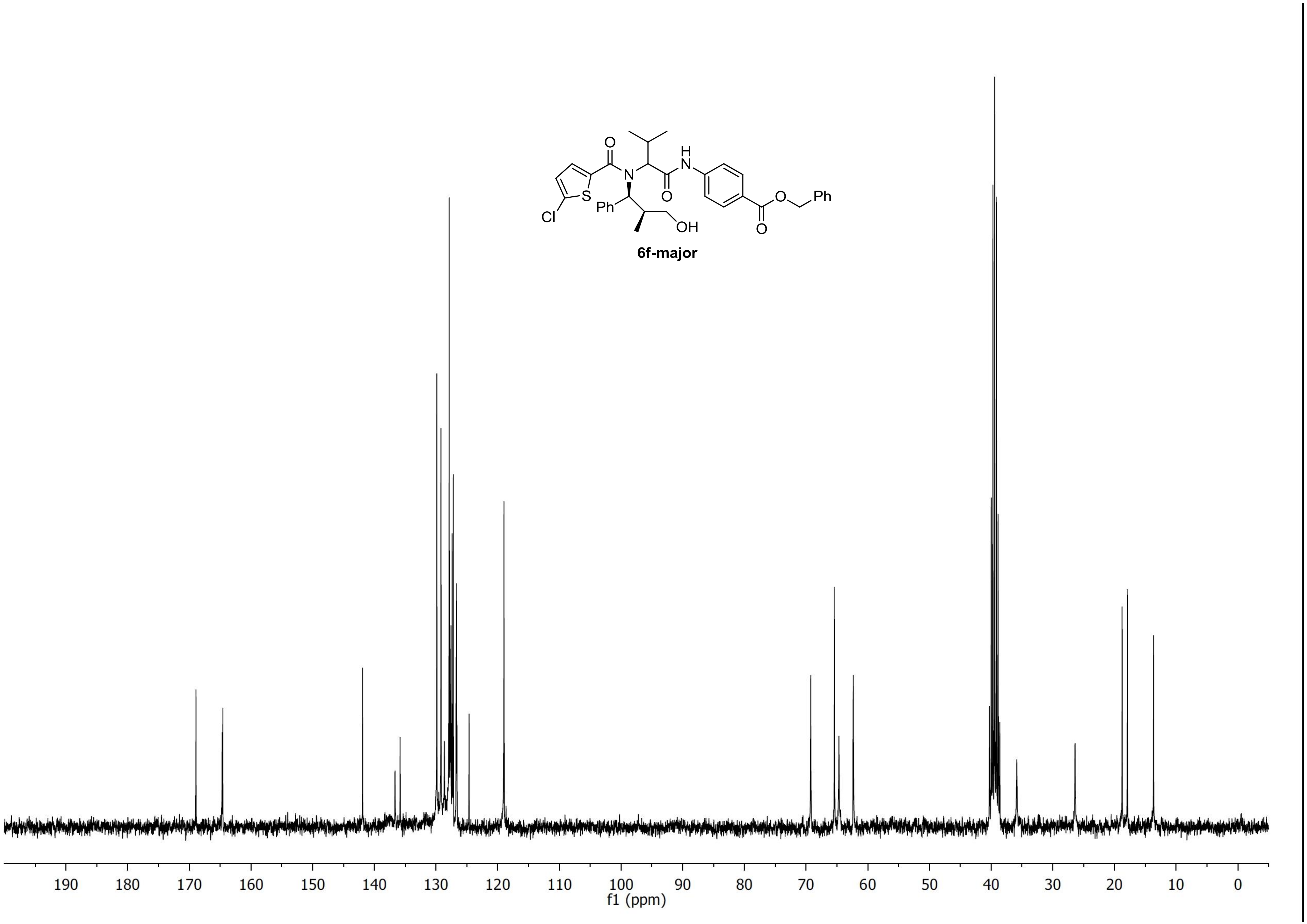
O
H
N
S
Cl
Ph
O
OH
O
Ph
O
6f-major
190 180 170 160 150 140 130 120 110 100 90 80 70 60 50 40 30 20 10 0
f1 (ppm)

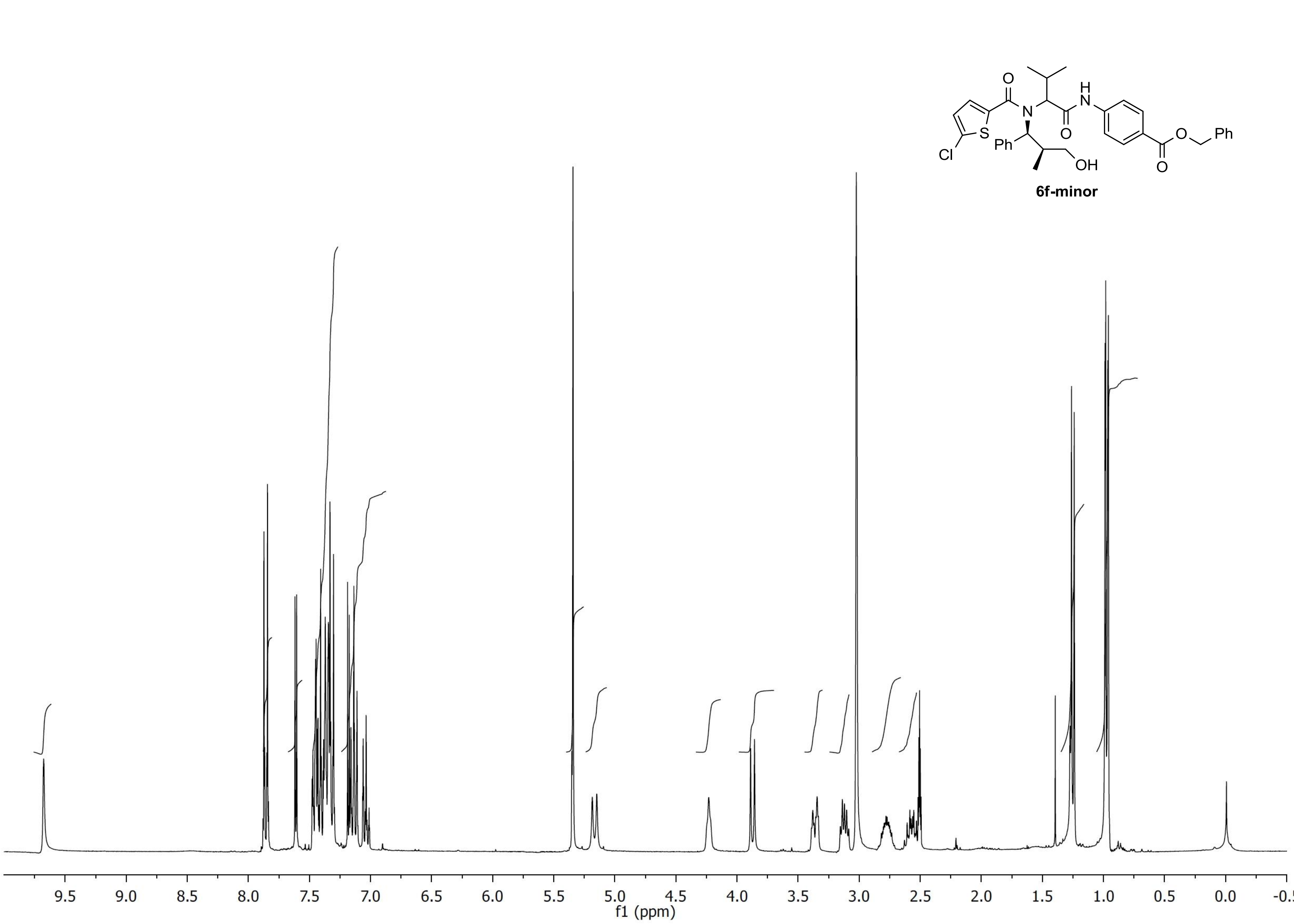
6f-minor
9.5
9.0
8.5
8.0
7.5
7.0
6.5
6.0
5.5
5.0
4.5
4.0
3.5
3.0
2.5
2.0
1.5
1.0
0.5
0.0
-0.5
f1 (ppm)

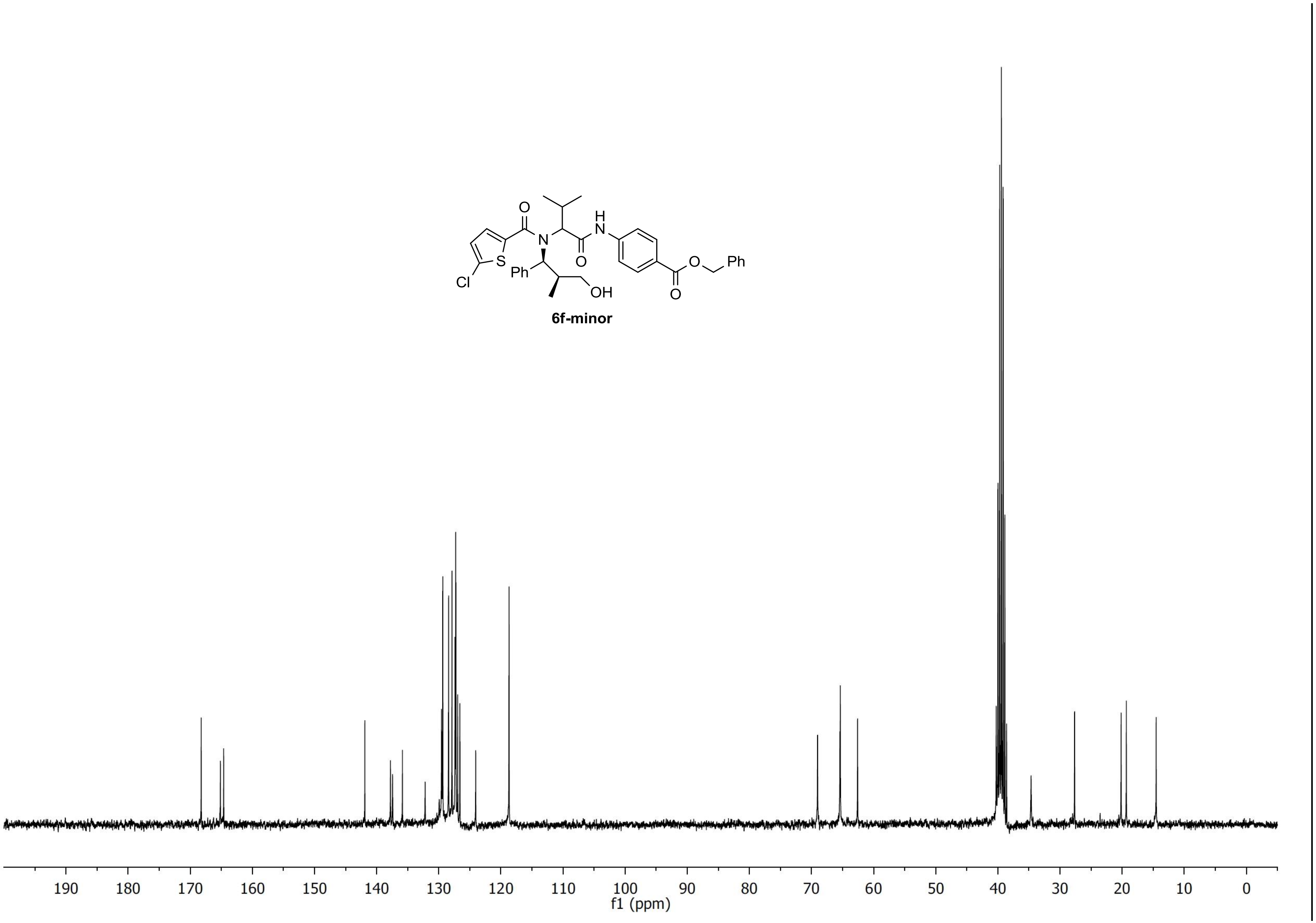
O
N
H
S
Cl
Ph
OH
O
O
O
Ph

6f-minor

190 180 170 160 150 140 130 120 110 100 90 80 70 60 50 40 30 20 10 0
f1 (ppm)

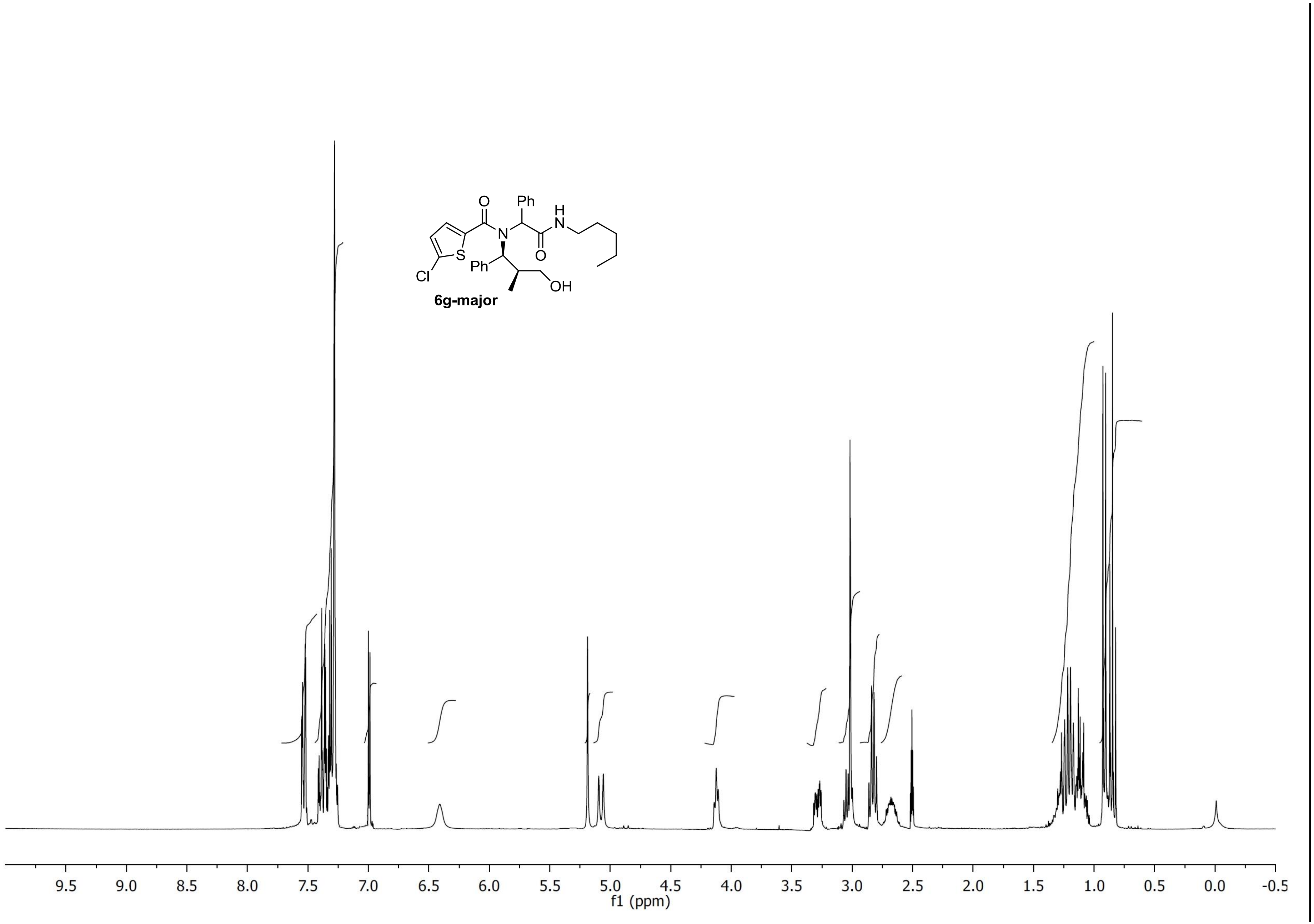

6g-major
f1 (ppm)

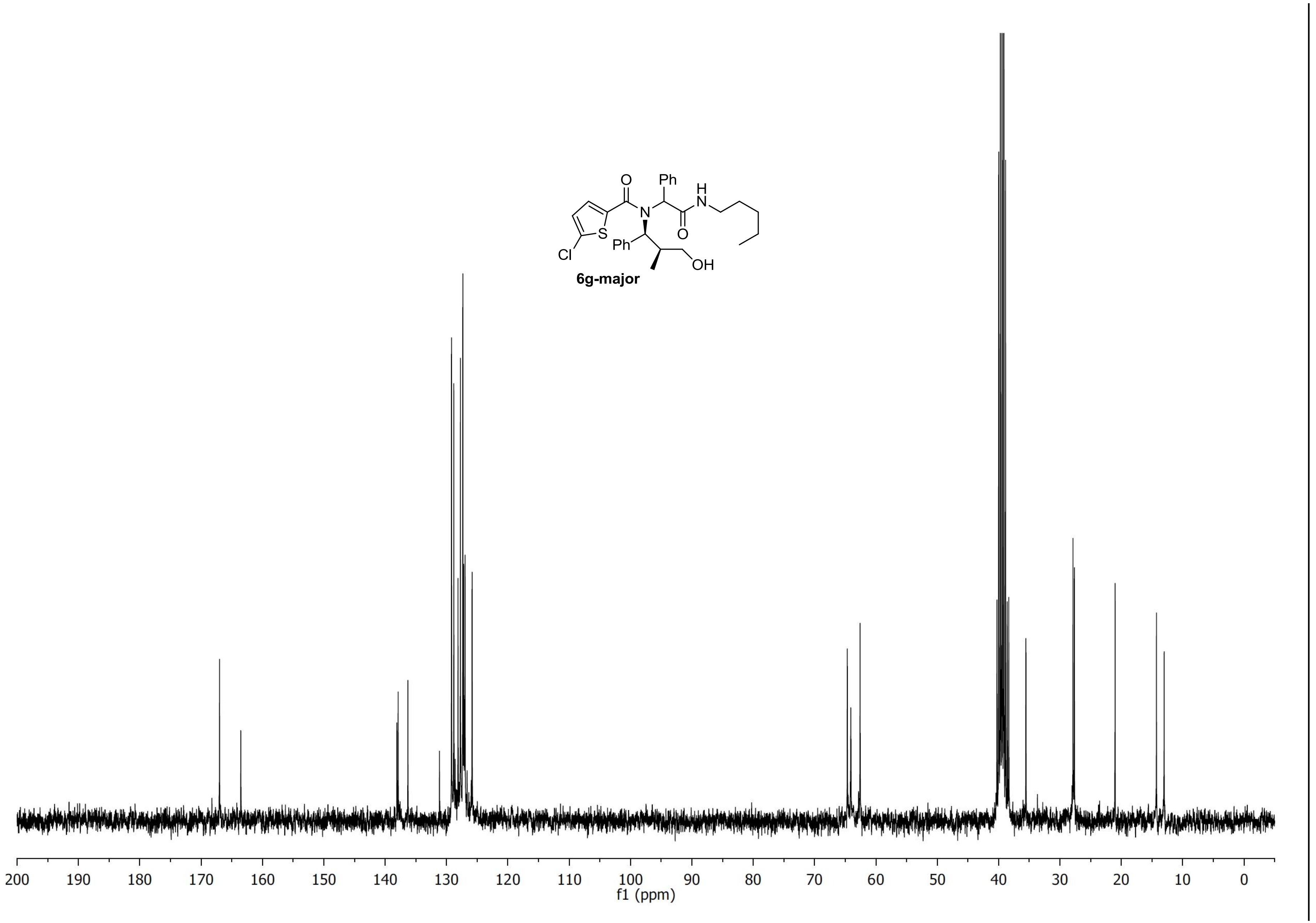
6g-major
Ph
Ph
Cl
S
N
O
O
H
N
OH
200 190 180 170 160 150 140 130 120 110 100 90 80 70 60 50 40 30 20 10 0
f1 (ppm)

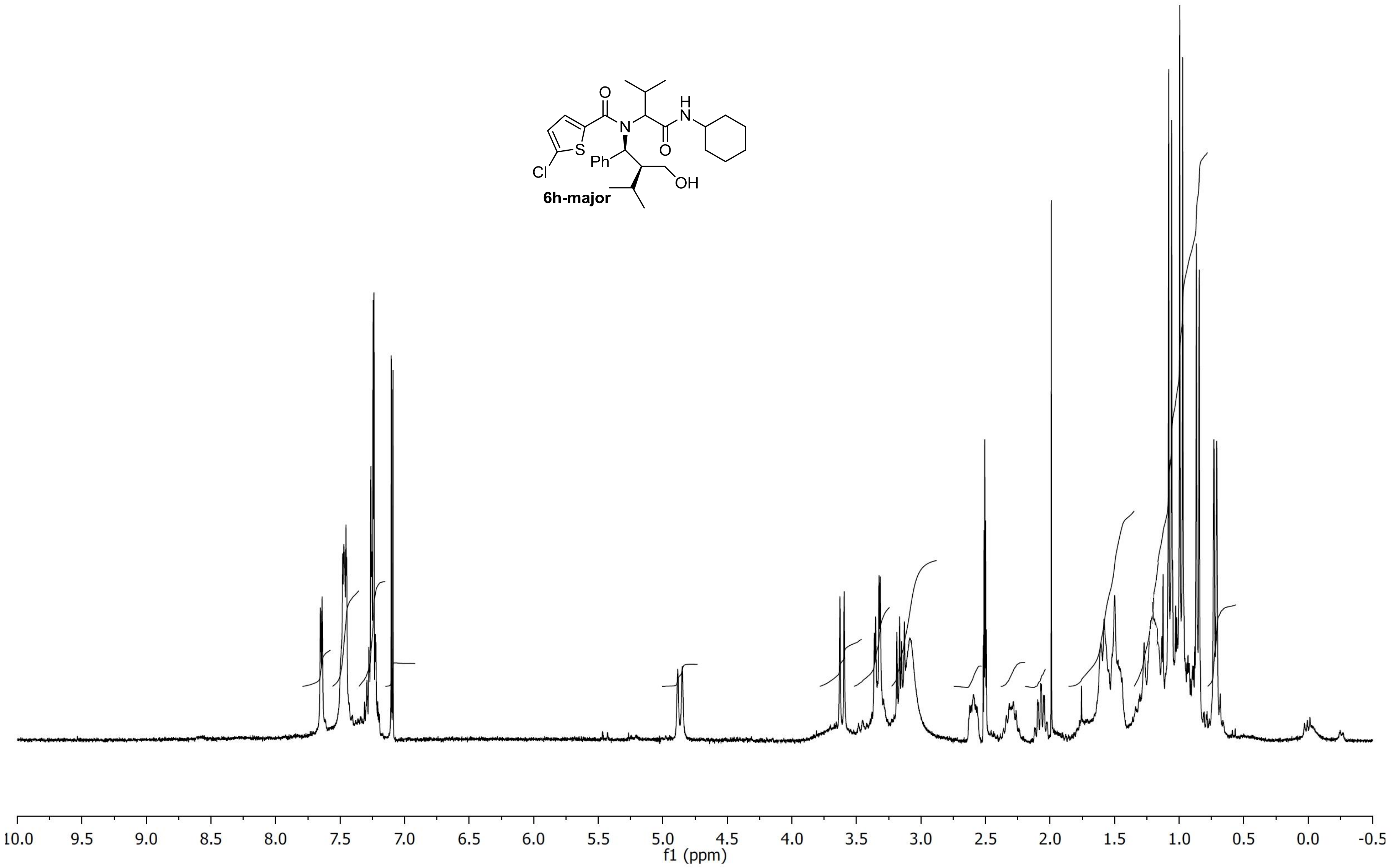
6h-major
10.0 9.5 9.0 8.5 8.0 7.5 7.0 6.5 6.0 5.5 5.0 4.5 4.0 3.5 3.0 2.5 2.0 1.5 1.0 0.5 0.0 -0.5
f1 (ppm)

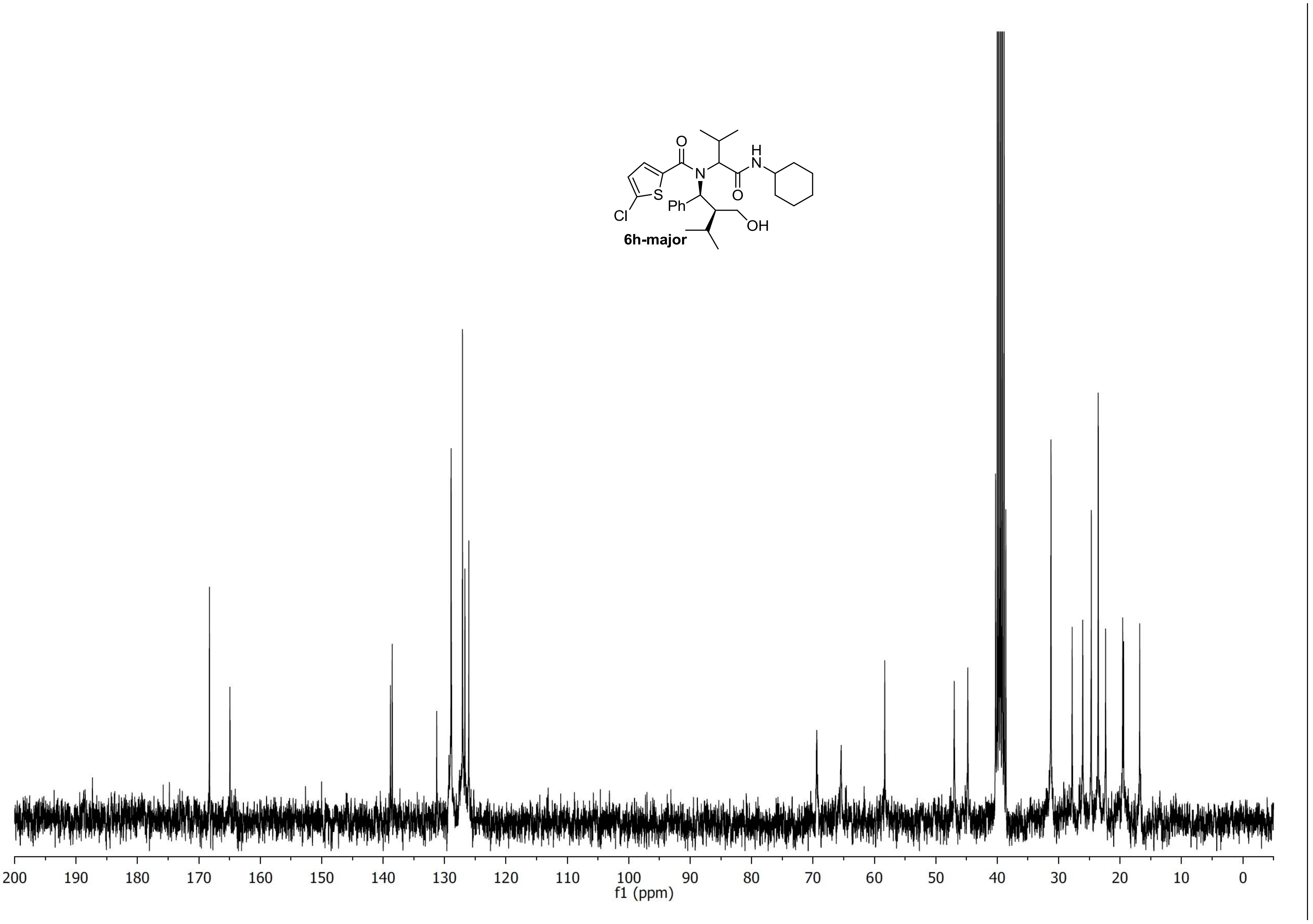

O
N
H
N
O
S
Cl
Ph
OH
6h-major
200 190 180 170 160 150 140 130 120 110 100 90 80 70 60 50 40 30 20 10 0
f1 (ppm)

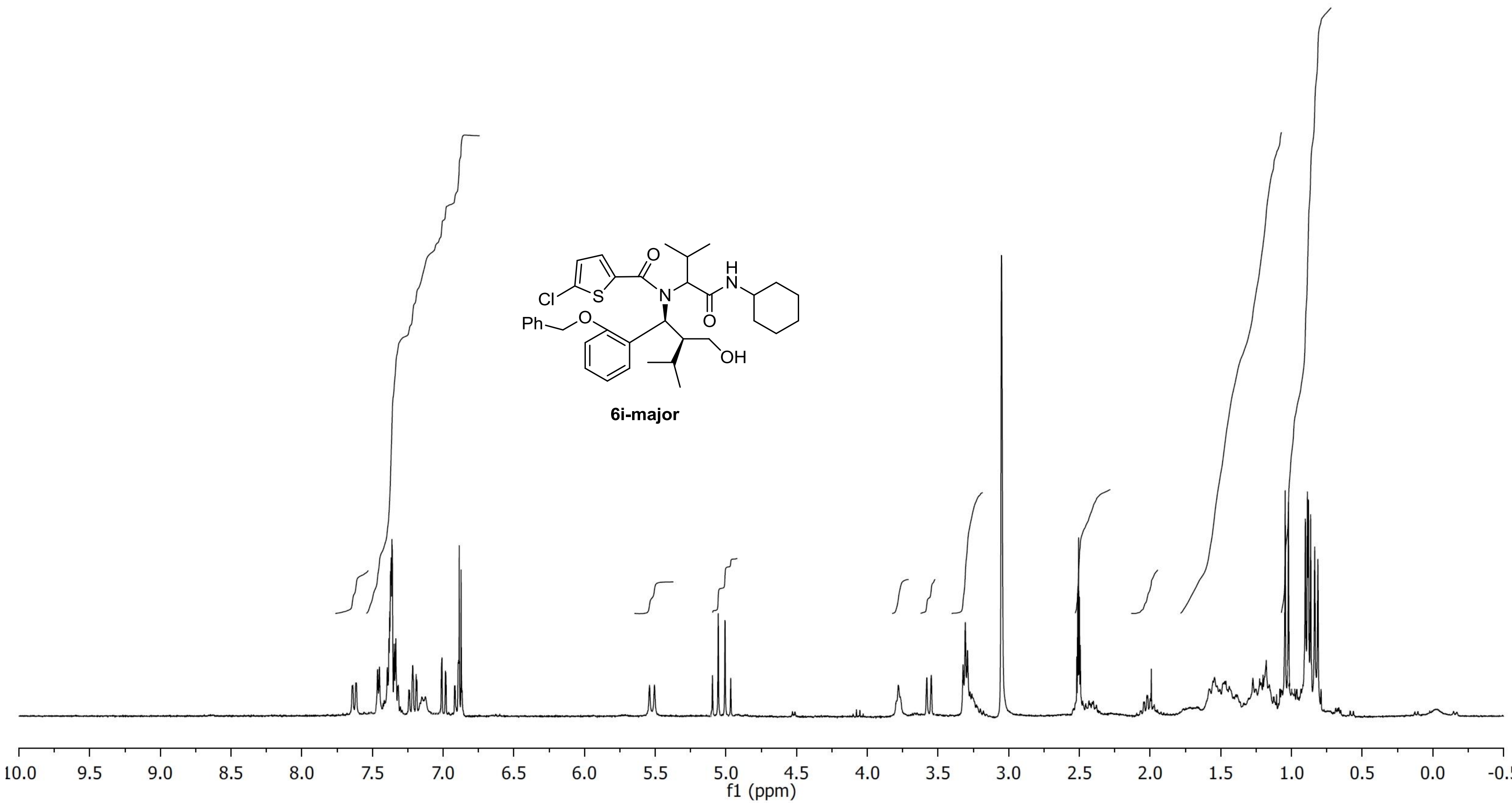
6i-major
10.0 9.5 9.0 8.5 8.0 7.5 7.0 6.5 6.0 5.5 5.0 4.5 4.0 3.5 3.0 2.5 2.0 1.5 1.0 0.5 0.0 -0.5
f1 (ppm)

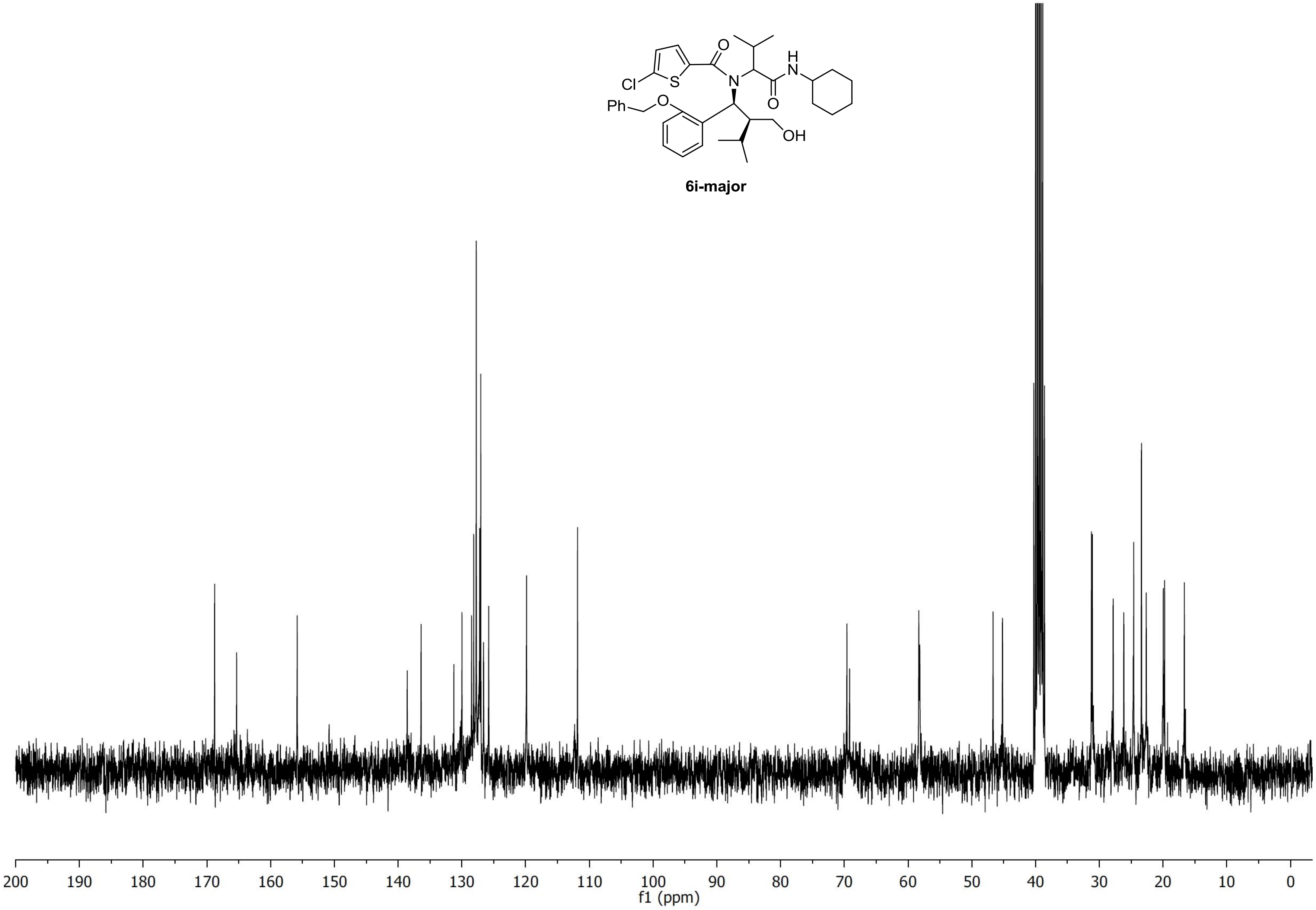
O
H
N
Cl
S
N
O
Ph
O
OH
200 190 180 170 160 150 140 130 120 110 100 90 80 70 60 50 40 30 20 10 0
f1 (ppm)

6i-major

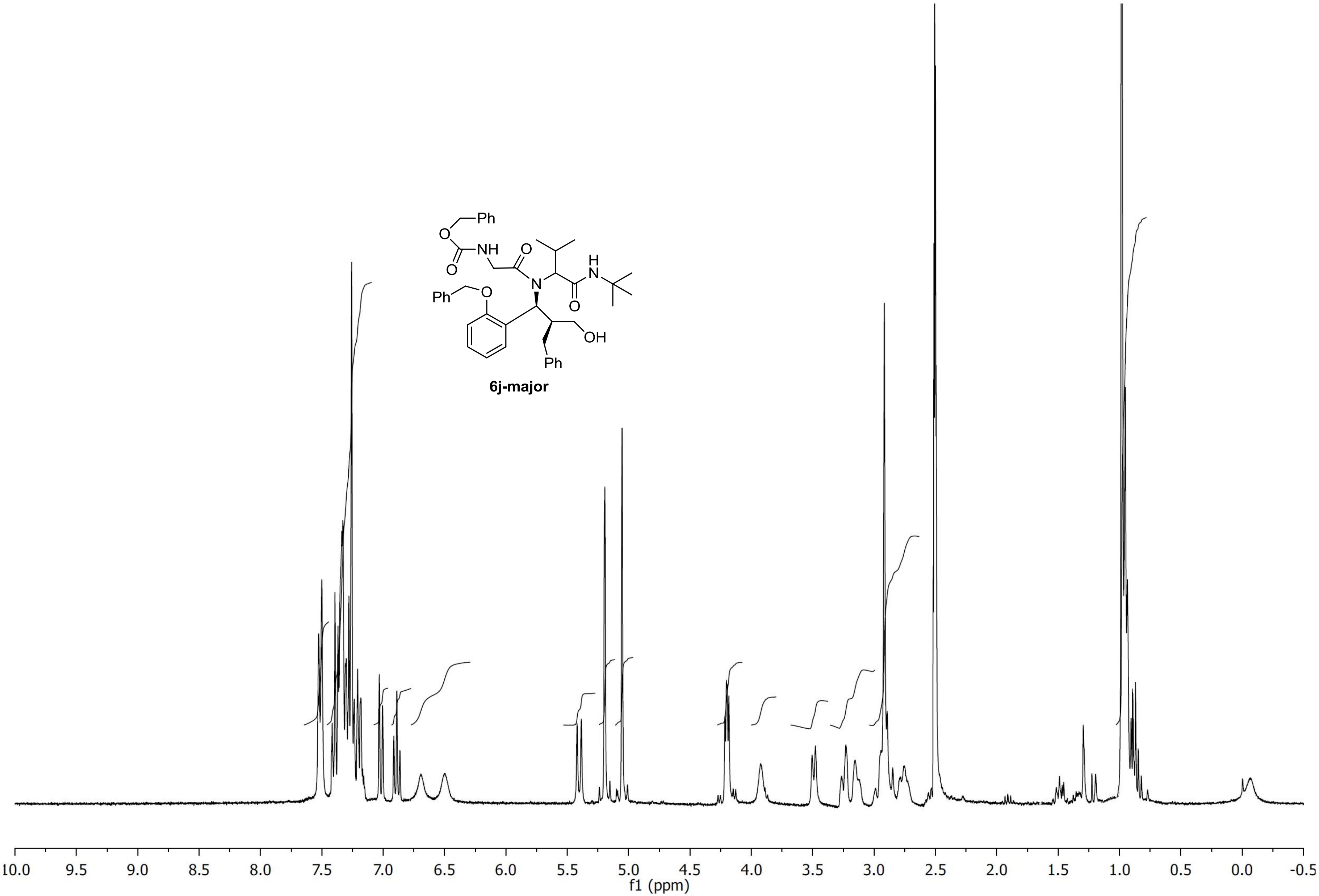
Ph
O
NH
O
O
N
H
N
O
Ph
O
OH
Ph
6j-major
10.0
9.5
9.0
8.5
8.0
7.5
7.0
6.5
6.0
5.5
5.0
4.5
4.0
3.5
3.0
2.5
2.0
1.5
1.0
0.5
0.0
-0.5
f1 (ppm)

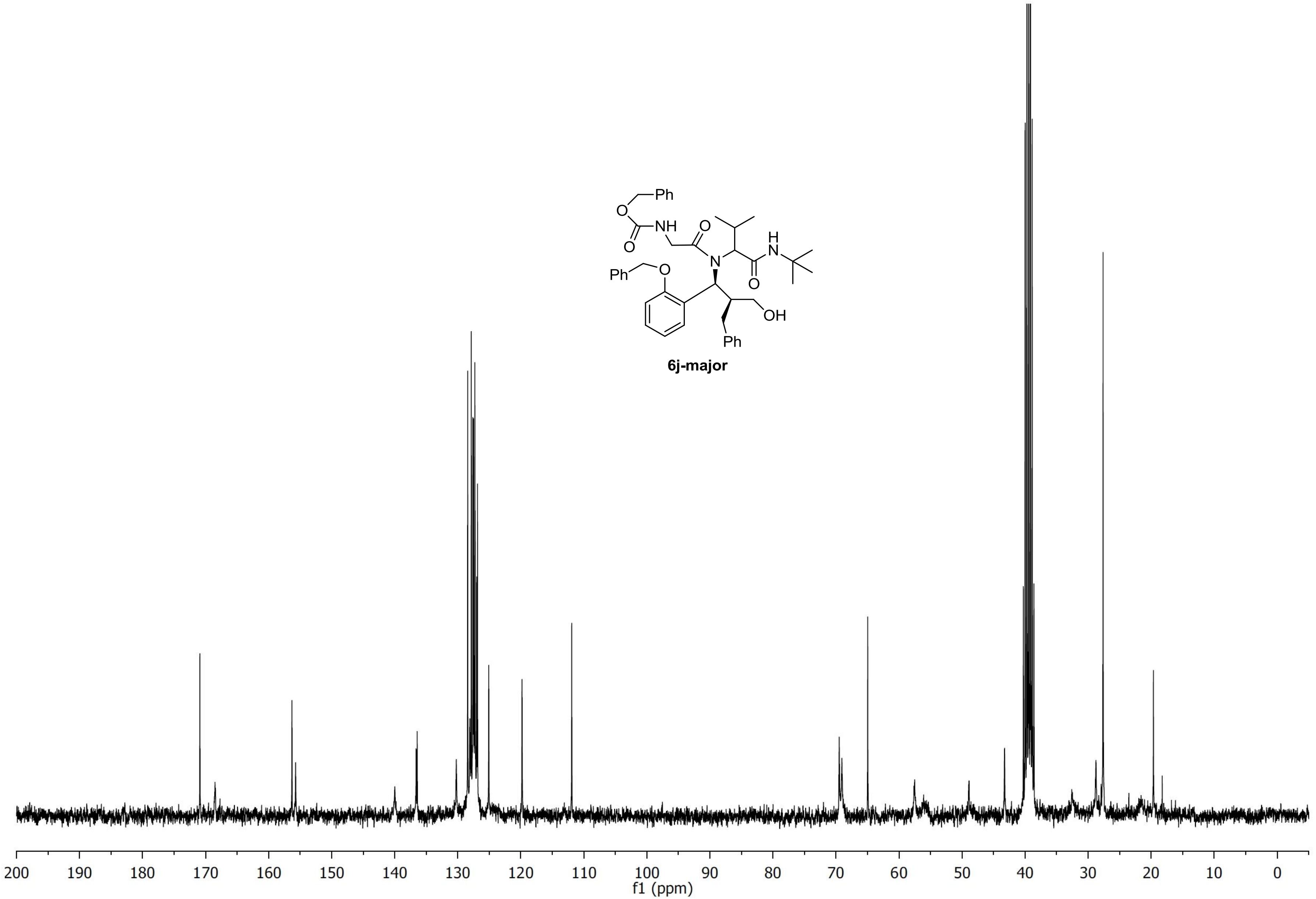
Ph
O
O
NH
O
N
H
N
O
Ph
O
OH
Ph
6j-major
200
190
180
170
160
150
140
130
120
110
100
90
80
70
60
50
40
30
20
10
0
f1 (ppm)
